# The invisible witness: air and dust as DNA evidence of human occupancy in indoor premises

**DOI:** 10.1038/s41598-023-46151-7

**Published:** 2023-11-04

**Authors:** Chiara Fantinato, Ane Elida Fonneløp, Øyvind Bleka, Magnus Dehli Vigeland, Peter Gill

**Affiliations:** 1 Department of Forensic Sciences, Oslo University Hospital, Oslo, Norway; 2 Department of Forensic Medicine, Institute of Clinical Medicine, University of Oslo, Oslo, Norway; 3 Centre for Ecological and Evolutionary Synthesis (CEES), Department of Biosciences, University of Oslo, Oslo, Norway

**Keywords:** Biological techniques, Computational biology and bioinformatics

## Abstract

Humans constantly shed deoxyribonucleic acid (DNA) into the surrounding environment. This DNA may either remain suspended in the air or it settles onto surfaces as indoor dust. In this study, we explored the potential use of human DNA recovered from air and dust to investigate crimes where there are no visible traces available—for example, from a recently vacated drugs factory where multiple workers had been present. Samples were collected from three indoor locations (offices, meeting rooms and laboratories) characterized by different occupancy types and cleaning regimes. The resultant DNA profiles were compared with the reference profiles of 55 occupants of the premises. Our findings showed that indoor dust samples are rich sources of DNA and provide an historical record of occupants within the specific locality of collection. Detectable levels of DNA were also observed in air and dust samples from ultra-clean forensic laboratories which can potentially contaminate casework samples. We provide a Bayesian statistical model to estimate the minimum number of dust samples needed to detect all inhabitants of a location. The results of this study suggest that air and dust could become novel sources of DNA evidence to identify current and past occupants of a crime scene.

## Introduction

A human adult sheds about 1000 skin cells per cm^2^ per hour, causing billions of cells to be released from the body every day^1,2^. This happens through epidermal desquamation, which is a continuous process that constitutes the final active step in the keratinocytes’ differentiation program. Desquamation causes a spontaneous detachment of dead skin cells known as corneocytes^2–4^. The average size of these skin particles is smaller than the pores of typical clothing fabrics, allowing them to pass through and become aerosolized^5,6^. Varying levels of DNA may be retained within corneocytes; enough to yield detectable profiles^7–10^. DNA can be released in the air not only through skin cells but also in other forms. Dandruff constitutes part of bioaerosol material shed by humans, with a single particle containing between 0.8 and 16.6 ng of DNA^11^. Detectable amounts of DNA can also be transferred to the surrounding environment by an individual speaking, coughing or sneezing^12–14^. The potential for indirect DNA transfer through agitation of dry biological materials, including DNA on touched surfaces, and personal items like clothing, has also been demonstrated in the absence of direct contact^15,16^. Individuals can deposit DNA on untouched surfaces in areas they occupy; the probability of detecting DNA increases with the duration of their presence^17^. Furthermore, significant differences were observed in the propensity of individuals to shed DNA into their immediate surroundings, without coming into direct contact with surfaces; an “environmental shedder status”^17^ can be assigned to individuals to quantify this variability. Skin cells and DNA fragments floating in the air make up a significant proportion of indoor dust^18,19^. It was previously demonstrated by Toothman et al.^19^ that human DNA is present in indoor dust in sufficient quantity and quality to produce allele calls in STR analysis. However, the work described in this paper is the first study that explores the potential use of air and dust to investigate serious crimes, along with a description of limitations and potential pit-falls.

There are many cases in police investigations that require identification of individuals from crime scenes^20^. Some examples are cases of burglaries, homicides, domestic violence and identification of members of illegal organizations. Often, the most difficult cases involve organized crime and terrorism, where the participants may be forensically aware, hence the detection of conventional DNA and fingerprints may be difficult to achieve. This happens when (a) there is no body fluid to identify, (b) surfaces such as tables, door handles, light switches may have been cleaned or (c) criminals may have worn gloves, which minimizes their direct skin contact with surfaces. 

In addition, criminals typically employ “safe-houses”, which may change over time to evade detection. These “safe-houses” serve as temporary locations for various illegal activities, ranging from the production of illicit drugs and explosives to housing illegal organizations such as cults, scams syndicates, and unlicensed clinics, often under clever disguises^21^. These activities, regardless of their nature, could potentially lead to police raids based on intelligence reports. During such operations, while some of the current occupants may be apprehended, it becomes essential to gather evidence not only from those currently present but also from individuals who may have been associated with these locations at some point in the past. To achieve this, DNA isolated from dust provides an historical record and may be useful to identify occupants over a long time-frame, from a few months up to a few years. Conversely, DNA extracted from air samples could provide a more recent record: collection of air samples may be useful in specific cases where the offender has been present in a confined area for a short period of time, insufficient for skin cells and DNA to accumulate in dust. For example, air could be a valuable source of information in the case of car theft, where the forensically aware criminal may take care to wear gloves, which reduces the possibility of DNA detection from surfaces such as steering wheels. 

Whereas, the investigation of crime is a strong motivation, it is important to consider the practical implications of collection and analysis of air and dust samples in the laboratory environment. The high sensitivity of DNA technology makes it possible to obtain results from very low levels of biological materials (just a few cells)^22,23^. Consequently, there is an increased possibility that environmental background DNA, defined as originating from unknown individuals^24^, may be detected^24–30^. In a laboratory where cases are analyzed, there is a risk that aerosol DNA may contaminate the evidence. Two different studies^30,31^ showed that air is unlikely to be a source of contamination in a forensic laboratory; however, the experiments were conducted more than 10 years ago, using methods less sensitive than those available today.

Dust and air may result in challenging forensic samples, typically consisting of DNA mixtures from multiple contributors, and prone to allelic dropout due to degraded DNA^32^. Moreover, the police may have an incomplete list of suspects with which to compare evidential samples. They may wish to extend the search to encompass national DNA databases of reference samples. This may entail many thousands or even millions of comparisons in order to properly search databases to compile lists of suspects, and to identify which evidential material they may have contributed to. Traditional database search engines such as CODIS (Combined DNA Index System)^33^ are inefficient, requiring user input, and limited to profiles that are well represented. An alternative strategy is to use probabilistic genotyping to calculate likelihood ratio statistics, where the value of the evidence is quantified for any given candidate donor to a questioned DNA profile. The open-source software EuroForMix^34^ is utilized for this purpose. Because of the considerable automation inherent with this program, it is highly efficient; here we provide a demonstration with a small reference database.

We have used the building of our institute as the test-bed for the investigation; the experiments were conducted in 14 offices, two meeting rooms and five laboratory areas which are accessed by corridors. Following ethical approval, a reference database was built from employees that gave written consent for their profiles to be included (n = 55 of 64 employees). This was used to compare findings from air and dust samples taken from all areas of the building. Laboratory areas were of particular interest, since it is here that risks of contamination of evidence samples must be carefully managed. Novel methods to collect, isolate, and analyze human DNA from the air and dust were developed for the study.

## Results

### Characterization of DNA profiles in air and dust samples

**Figure 1:**
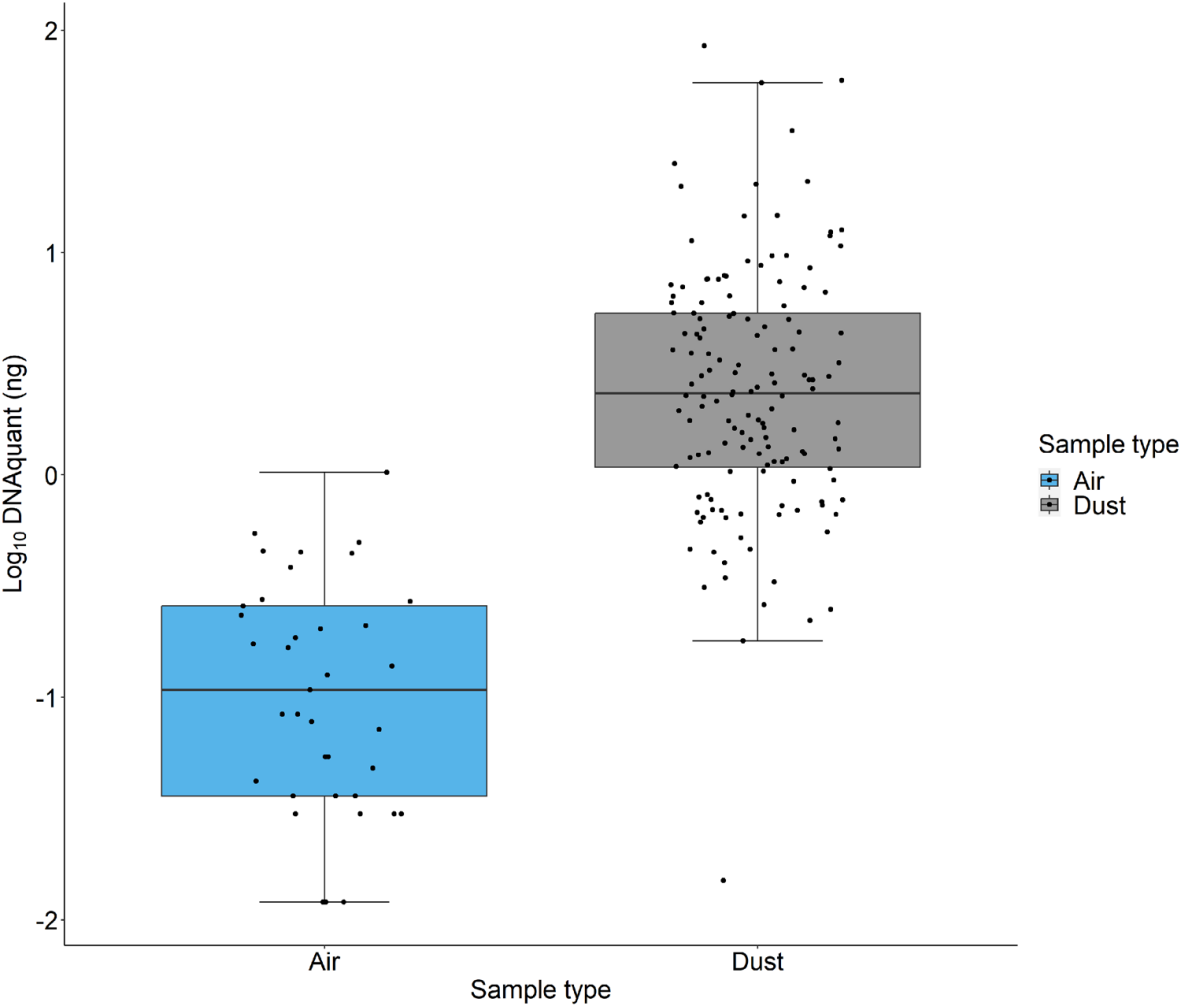
Log_10_ of DNA quantity (ng) recovered for air and dust samples.

A total of 40 air samples and 144 dust samples were collected and analyzed from 14 offices, two meeting rooms and five laboratory areas. Air samples displayed significantly lower DNA quantities (median = 0.1 ng) compared to dust samples (median = 2.3 ng), (Wilcoxon, p < 2.2 × 10^–16^), (Fig. 1). For detailed information on each sample, refer to Supplementary Data 1.

**Figure 2:**
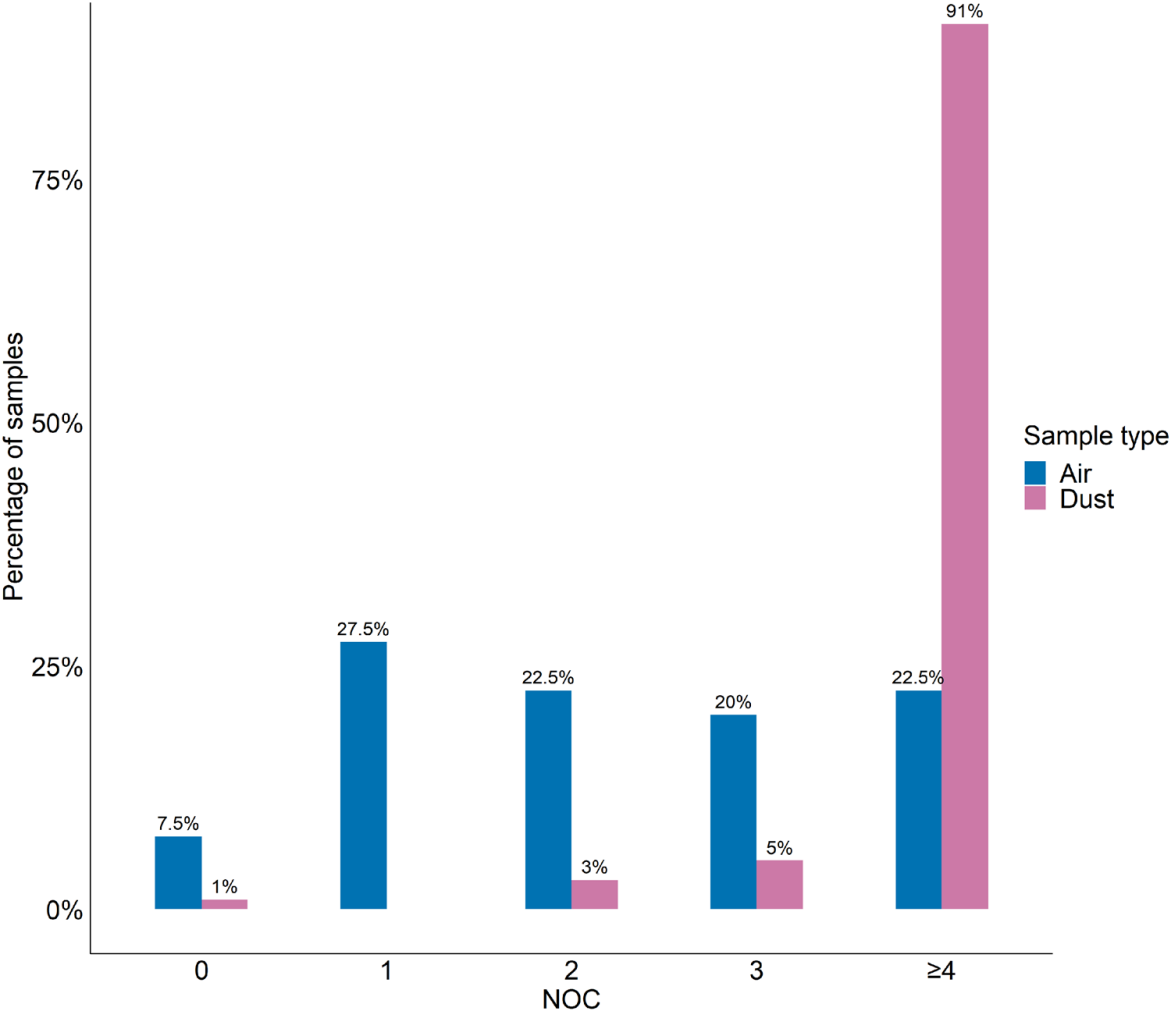
Distribution of the number of contributors (NOC) detected in air and dust samples.

Among the air samples, 7.5% (N = 3) did not provide any DNA profile, 27.5% (N = 11) resulted in partial single-source profiles, whilst 65% (N = 26) produced DNA mixtures with varying numbers of alleles detected. Two-, three-, and four-person mixtures were observed in approximately equal proportions (20%-22.5%). The dust samples generally exhibited more complex mixtures than air samples, with 91% (N = 131) of the dust samples indicating four or more contributors. Four was the maximum number of contributors (NOC) considered in the analysis; the threshold was applied to the settings in the dnamatch2 software, which was used to compare resultant DNA profiles to reference samples of the participants of this study (see Methods): more could be considered but the analysis time increases exponentially. Only 3% (N = 5) of the dust samples resulted in two-person mixtures, whilst 5% (N = 7) resulted in three-person mixtures (Fig. 2).

A reference database $R$ was prepared of 55 out of 64 employees at our institute. To detect ‘matches’ with any given air and dust sample, a likelihood ratio (LR) was calculated (Eq. (1)) for each reference sample (*PoI*) in the database, where for a given evidence sample (*E*), the following hypotheses were compared:

1 $$LR=\frac{\mathrm{Pr}\left(E|{H}_{1}\right)}{\mathrm{Pr}\left(E|{H}_{2}\right)}$$

Likelihood ratios (Eq. (1)) were calculated by comparison of two alternative propositions (*H*_*1*_) and (*H*_*2*_). Under ${H}_{1}$ the reference profile of a given person of interest (*PoI)* is conditioned, which is an individual from the reference database $R$ (see Methods for further details) and there are *L-1* unrelated unknown contributors. Under* H*_*2*_ the *PoI* is replaced by an unknown contributor. The *LR* is summarized: ${H}_{1}$: “$PoI$ and $L-1$ unrelated unknowns are contributors” versus ${H}_{2}$: “$L$ unrelated unknowns are contributors”.

The expected number of false positives when searching a database of random individuals, based on a “positive” criterion $LR\ge x$ where $x$ is a specified threshold, can be derived from the Turing expectation $p\left(LR > x|Hd\right)<\frac{1}{x}$^35–37^. If a database is of size *N* we never expect more than $N/x$ adventitious matches. It follows that if $N$ can be reduced by searching a subset of a database, e.g., by searching for individuals of a certain age-range or location, this will usefully reduce the chance of non-contributor matches. This does not mean that all positives are true positives, neither does it mean that all negatives are true negatives. All database search strategies are investigative rather than evaluative. The aim is to provide the authorities with a list of potential suspects that may be further investigated. If the profile is poorly represented then a *PoI* may be present, but undetected because the LR is below threshold. Where LRs are above threshold, the *PoI* may be further investigated, but the aim is to minimize the number of adventitious matches rather than to avoid them completely. 

For this study we used an arbitrary LR = 100 as a reasonable threshold to distinguish a positive (a match) from a negative result. If $N$=1mill, then the LR threshold $x$ would need to be equivalent in size, to avoid too many adventitious matches. The analysis returns a list of candidates for further investigation. The resulting LR values ranged from 1 to 3.78 × 10^32^, for 55 *PoIs* from each of the evidence samples that gave a DNA profile.

**Figure 3:**
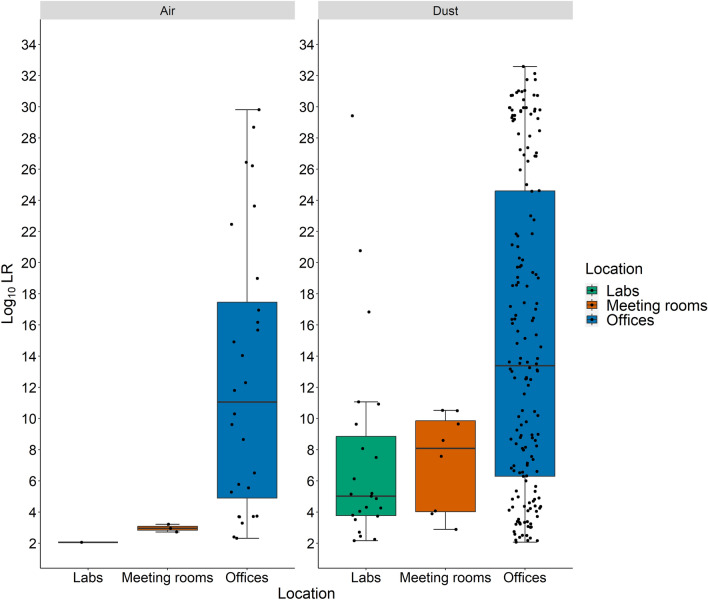
Log_10_LR values (LR ≥ 100) for air and dust samples collected from three different locations (laboratories, meeting rooms and offices).

Comparing the log_10_LR distributions of those samples that provided positive matches (LR ≥ 100), from the different types of sampling locations (Fig. 3), showed that offices generally gave higher log_10_LR values than meeting rooms and laboratories, both for air and dust samples. Higher LRs are associated with higher quality of the DNA profiles obtained. For the air samples, the log_10_LR values were significantly different between offices and meeting rooms (Wilcoxon, *p* = 0.03). Only one air sample from laboratories gave LR > 100 (Fig. 3). Similarly, for dust samples, log_10_LR values from offices were higher than for meeting rooms, at a level approaching statistical significance (Wilcoxon, *p* = 0.06) and significant for laboratories (Wilcoxon, *p* = 9 × 10^–4^). No significant differences were observed between meeting rooms and laboratories (Wilcoxon, *p* = 0.5). The log_10_LR values for each sample are given in Supplementary Data 2 and 3.

## Success rate of identifying known occupants and non-occupants

A given evidence sample may contain DNA from both known and unknown contributors, else the result may be negative. All office occupants, except two (from offices R4 and R12), participated in the study and had their reference profile included in the reference database used for comparison in the dnamatch2 software^38^. Success rates were defined as the proportion of samples where one or more known occupants (*φ*_*1*_) and non-occupants (*φ*_*2*_) were recovered, regardless of whether each individual was detected only in one sample (unique observation) or more; calculated using Eq. (4) and Eq. (5) in “Methods”. These parameters were calculated for dust and air samples collected from the three different locations: offices, meeting rooms and laboratories (divided into washing room, DNA casework laboratories and investigation room). The success rate is a convenient way to compare the results from samples collected from different environments. The results from all experiments are summarized in Table 1.

**Table 1:** Success rates for samples collected from the three different locations (offices, meeting rooms and laboratories).

Location (z)	${S}_{z}$	No. positive samples occupants	${N}_{z}$	No. positive samples non-occupants	${NC}_{z}$	*φ*_*1*_	*φ*_*2*_
Dust							
Offices	96	93	3	22	74	0.97	0.23
Meeting rooms	24	7	17	–	–	0.29	–
Washing room	8	7	1	2	6	0.88	0.25
DNA casework laboratories	12	7	5	2	10	0.58	0.17
Investigation room	4	2	2	0	4	0.50	0
Air							
Offices 0 h	17	12	5	1	16	0.71	0.06
Offices 16 h	8	2	6	4	4	0.25	0.5
Meeting rooms	6	3	3	–	–	0.50	–
Washing room	2	1	1	0	2	0.50	0
DNA casework laboratories	3	0	3	0	3	0	0
Investigation room	1	0	1	0	1	0	0

${S}_{z}$: number of samples taken from location *z*; No. positive samples occupants: number of samples where at least one known occupant was detected; ${N}_{z}$: number of samples from location *z* where no known occupants were positively identified, including samples with negative results (no DNA recovered/no matches with LR > 100); No. positive samples non-occupants: number of samples where at least one known non-occupant was detected. ${NC}_{z}$: number of samples from location* z* where no known non-occupants were positively identified, including samples with negative results (no DNA recovered/no matches with LR > 100). $\varphi$_1_: success rate for occupants, $\varphi$_2_: success rate for non-occupants. Offices 0 h: includes air samples collected 0 h after occupancy; Offices 16 h: includes air samples collected 16 h after occupancy.

### DNA recovery from dust

A total of 96 dust samples were collected across 14 different offices (1–7 occupants). All samples gave DNA results, however three were negative as they did not give any match with a known *PoI* with LR > 100, (Table 2). Positive results were obtained from 93 out of 96 dust samples for one or more office occupants, resulting in a success rate of *φ*_*1*_ = 0.97. Only 22 of the same samples matched with a known non-occupant, giving a non-occupant success rate of *φ*_*2*_ = 22/96 = 0.23.

**Table 2:** Data from dust samples collected from offices.

Location (z)	${K}_{z}$	No. untyped occupants	${S}_{z}$	${N}_{z}$	P1	P2	P3	P4	P5	P6	P7	No. unique occupants detected	No. unique non-occupants detected
R1	4	0	12	0	9	6	6	5	–	–	–	4	0
R2	5	0	12	0	11	7	2	0	0	–	–	3	1
R3	1	0	8	0	8	–	–	–	–	–	–	1	4
R4	4	1	8	0	8	7	2	–	–	–	–	3	1
R5	1	0	12	0	12	–	–	–	–	–	–	1	1
R6	3	0	8	1	7	1	0	–	–	–	–	2	3
R7	3	0	8	0	8	8	2	–	–	–	–	3	1
R8	3	0	4	0	4	3	0	–	–	–	–	2	0
R9	2	0	4	0	4	4	–	–	–	–	–	2	2
R10	7	0	4	0	3	1	1	0	0	0	0	3	1
R11	2	0	4	0	3	1	–	–	–	–	–	2	1
R12	4	1	4	0	4	3	0	–	–	–	–	2	2
R13	2	0	4	0	4	1	–	–	–	–	–	2	1
R14	1	0	4	2	2	–	–	–	–	–	–	1	1
Totals:	42	2	96	3								31	19

*K*_*z*_*:* number of unique known occupants in location *z;* No. untyped occupants: number of known occupants not participating in the study whose reference profile was not included in the reference database. ${S}_{z}$: total number of samples collected and analyzed from each location. *N*_*z*_: number of samples from location *z* where no known occupants were positively identified, including samples with negative results (no DNA recovered/no matches with LR > 100). P1,…,P7: number of times a known unique occupant is detected per location (z).

The results for meeting rooms and laboratories are detailed in Supplementary Tables 1, 2. For meeting rooms, the success rate for occupants was *φ*_*1*_ = 0.29, significantly lower than for offices *φ*_*1*_ = 0.97, (Fisher exact two tail test,* p* = 1.7 × 10^−12^). Meeting rooms are used by all 64 members of staff (albeit more sporadically than offices), hence, assuming each has equal probability of detection, the detection rate per member of staff is much lower than for offices (Supplementary Table 1).

Laboratories have restricted access to individuals who wear protective clothing in order to prevent cross-contamination of live casework material, so there is an expectation that recovery of DNA in dust and air should be lower than for other areas. In the washing room an individual (participant P1) was identified in seven out of eight dust samples collected from different surfaces; the success rate of detecting one or more occupants was surprisingly high *φ*_*1*_ = 0.88 but was influenced by presence of just one high-shedder-status individual found in multiple samples. However, two participants that had no regular access to the washing room were also detected; the success rate of detecting non-occupants in the washing room was calculated as *φ*_*2*_ = 0.25 (Supplementary Table 2).

A total of 12 dust samples were collected from DNA casework laboratories, 11 of these provided DNA results. The occupant success rate was *φ*_*1*_ = 0.58, however, this was also driven by participant P1 who was identified in five out of 12 samples collected. Two individuals who did not regularly work in the DNA laboratories, but declared that they had entered the rooms at least once, were also identified, giving a success rate for non-occupants of *φ*_*2*_ = 0.17 (Supplementary Table 2).

Two out of four samples collected from the investigation room provided a positive result for one or more occupants *φ*_*1*_ = 0.5. No known non-occupants were detected (Supplementary Table 2).

### DNA recovery from the air

The analysis of samples from the air required specialized equipment (see Methods) and collections took several hours, hence compared with analysis of dust samples, it was not as convenient. Consequently, the number of samples was restricted by the availability of sampling devices and data collections were not as extensive. 

For offices, the sampling regime was (a) 0 h after occupancy, where collection was carried out immediately following vacation of the room and (b) 16 h after occupancy. Two samples were collected at three days, and one sample was taken at 15 days after occupancy; these three samples were however excluded from the following analysis as the sample size was not representative of the two time-points. There was to our knowledge no movement into the room during sampling and doors were kept closed. All 17 samples collected from offices immediately after occupancy provided a DNA result. However, five samples did not provide any match with an LR > 100 (Table 3).

**Table 3:** Data from air samples collected from offices at 0 h after occupancy.

Location (*z*)	${{\varvec{K}}}_{{\varvec{z}}}$	No. untyped occupants	${{\varvec{S}}}_{{\varvec{z}}}$	${{\varvec{N}}}_{{\varvec{z}}}$	P1	P2	P3	P4	P5	P6	P7	No. unique occupants detected	No. unique non- occupants detected
R1	4	0	2	1	1	0	0	0	–	–	–	1	1
R2	5	0	2	0	2	0	0	0	0	–	–	1	0
R4	4	1	2	1	1	0	0	–	–	–	–	1	0
R5	1	0	1	0	1	–	–	–	–	–	–	1	0
R6	3	0	2	0	2	0	0	–	–	–	–	1	0
R7	3	0	2	0	2	2	0	–	–	–	–	2	0
R8	3	0	1	1	0	0	0	–	–	–	–	0	0
R9	2	0	1	0	1	0	–	–	–	–	–	1	0
R10	7	0	1	1	0	0	0	0	0	0	0	0	0
R12	4	1	1	0	1	0	0	–	–	–	–	1	0
R13	2	0	1	1	0	0	–	–	–	–	–	0	0
R14	1	0	1	0	1	–	–	–	–	–	–	1	0
Totals:	39	2	17	5								10	1

*K*_*z*_*:* number of unique known occupants in location *z;* No. untyped occupants: number of known occupants not participating in the study whose reference profile was not included in the reference database. ${S}_{z}$: total number of samples collected and analyzed from each location. *N*_*z*_: number of samples from location *z* where no known occupants were positively identified, including samples with negative results (no DNA recovered/no matches with LR > 100). P1,…,P7: number of times a known unique occupant is detected per location (z).

In office samples, occupant success rate for the 0 h samples (*φ*_*1*_ = 0.71) was comparable to dust, while the non-occupant success rate was much lower (*φ*_*2*_ = 0.06), (Table 3). For 16 h samples, occupants’ *φ*_*1*_ = 0.25 was lower than for 0 h samples (Fisher exact test p = 0.08) and was exceeded by non-occupants’ *φ*_*2*_ = 0.50 (Fisher exact test, *p* = 0.02), (Supplementary Table 3). In addition, LR values were found to be lower for samples collected 16 h after occupancy in comparison to samples collected immediately after occupancy, and this is a reflection of reduced quality of DNA recovery (Supplementary Data 2).

 Three individuals were identified from air samples collected from meeting rooms, giving a success rate of *φ*_*1*_ = 0.5. Participant P1 was one of the three individuals identified (Supplementary Table 4).

In laboratories, only one positive recovery was recorded. The air sample was collected from the washing room L1, it consisted of very low quality DNA, with an LR = 100 (Supplementary Table 5).

## Probability of detecting an occupant in a sample relative to the number of occupants in a location

**Figure 4:**
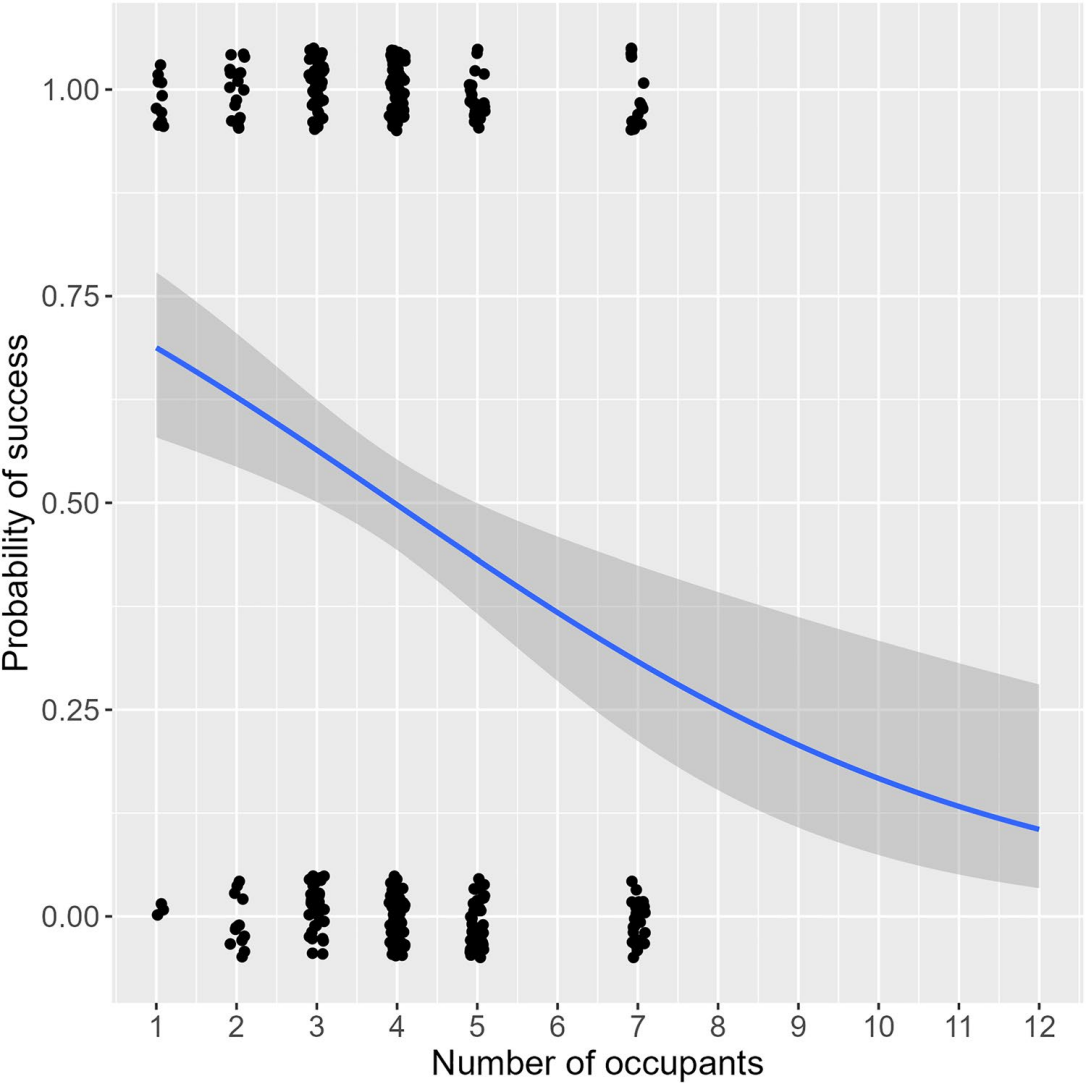
Logistic regression of dust samples from offices, comparing number of occupants in a given office vs probability of detection (success) of any given occupant in any given sample analyzed from that office. The blue line represents the logistic regression model fitted to the data and the grey area is the 95% confidence interval. Hosmer Lemeshow goodness of fit test gave p-value = 0.43, indicating a good fit to the data.

We estimated the probability of detecting DNA of an occupant in a location with *K* number of occupants. For this we used logistic regression based on the observed number of successes per occupant, per office, for dust samples presented in Table 2. The logistic regression (Fig. 4) assumes $${X}_{k,z}$$~Binom($${S}_{z},{p}_{K})$$, where $${X}_{k,z}$$ is the number of positive samples where an occupant $$k$$ in a location *z* with *K* occupants was detected such that the estimated probability becomes:

2 $${p}_{K}=\frac{1}{1+{e}^{-(b0+b1K)}}$$

with estimated coefficients as ${b}_{0}=1.06$ (intercept) and ${b}_{1}=-0.27$ (slope).

As displayed in Fig. 4, there is an inverse relationship between the number of occupants (*K*) and the probability of detecting DNA from a given occupant *k*_*z*_; as *K* increases the probability trends towards zero.

There were insufficient data to carry out a meaningful logistic regression analysis of dust samples from meeting rooms and laboratories.

## Estimating the success rate of identifying all occupants of a location

We consider here the probability ${\upphi }_{\mathrm{z}}$ of observing all *K*_*z*_ occupants at least once, in a given location (*z*), amongst *S*_*z*_ samples that were collected. The formula is given below (Eq. (3)):

3 $${\upphi }_{z}= \prod\nolimits_{{k = 1..K_{z} }} {1 - \left( {1 - p_{{k,z}} } \right)^{{S_{z} }} }$$

Here ${\mathrm{p}}_{k,z}$ is the probability of detection of occupant *k* out of *K*_*z*_ occupants in a given location* z*, and is inferred using data from Table 2. With a frequentist approach, as an example, occupant *k* = P4 from office *z* = R1, there are ${X}_{k,z}=5$ out of *S*_*z*_ = 12 samples, which gives ${p}_{P4,R1}=\frac{{X}_{\mathrm{P}4,z}}{Sz}=5/12=0.42$

Because of limited number of observations, we continued with a Bayesian approach, which requires the definition of a prior distribution for the $${\mathrm{p}}_{k,z}$$ probabilities; a prior beta distribution $$B$$($$\alpha$$=3, $$\beta$$=3) was used for all probabilities. From this we generated posterior samples of $${\mathrm{p}}_{k,z}$$ for each occupant $$k$$ in location $$z$$, to obtain the posterior distribution of $${\upphi }_{\mathrm{z}}$$ (for given samples size $${S}_{z}).$$ The assumed prior can be justified from the logistic regression model in Fig. 4, which indicates the prior belief that the probability of success is a mean of 0.5 for the range of 1–7 occupants. This prior can be interpreted as being moderately informative but allows for a wide range of possible values to take account of shedder and occupancy status. Continuing with the example above, we obtain the posterior expectation as:

$$E({p}_{k,z})=\frac{{X}_{k,z}+\alpha }{{S}_{z}+\alpha +\beta }=\frac{5+3}{12+3+3}=0.44$$

Hence, we see that the prior has large influence on the expectation when the number of samples ${S}_{z}$ is low, and only small influence when the number of samples is high.

From the perspective of the investigator, it is important to estimate the number of dust samples (*S*_*z*_) that should be retrieved from a crime scene in order to have a reasonable probability to detect each of the *K* occupants of a location. To obtain different measures of central tendency to express the results, three different estimators of ${\upphi }_{\mathrm{z}}$ were employed across a range of values for *S*_*z*_: posterior expectation (EXP), posterior mode (MODE), and posterior medians (MEDIAN) were calculated based on the Bayesian approach.

**Figure 5:**
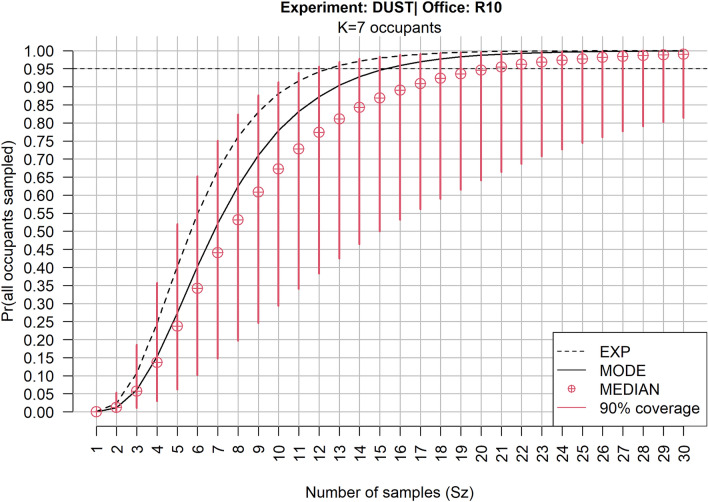
Office R10, *K* = 7: Plot of probability of detecting all *k*_*z*_* (*${\upphi }_{\mathrm{z}}$) at least once out of *K* occupants, in a set of samples, varying size (*S*_*z*_) using three different estimators.

The horizontal dashed line in Fig. 5 represents the 95% probability of obtaining all *K* occupants from *S*_*z*_ sample trials. Using the median estimate for example, this limit is achieved at *S*_*z*_ = 20. The wide coverage intervals indicates that the uncertainty of this estimate is very high. Other point estimates obtained a smaller number of trials. A higher confidence in the result would require a considerable higher number of trials.

The results for all offices are compiled in Supplementary Table 6 and Supplementary Figs. 1–13. The resulting figures show that the probability of identifying all occupants at least once is dependent on the total number of occupants in the location.

## Representing results by a match network

**Figure 6:**
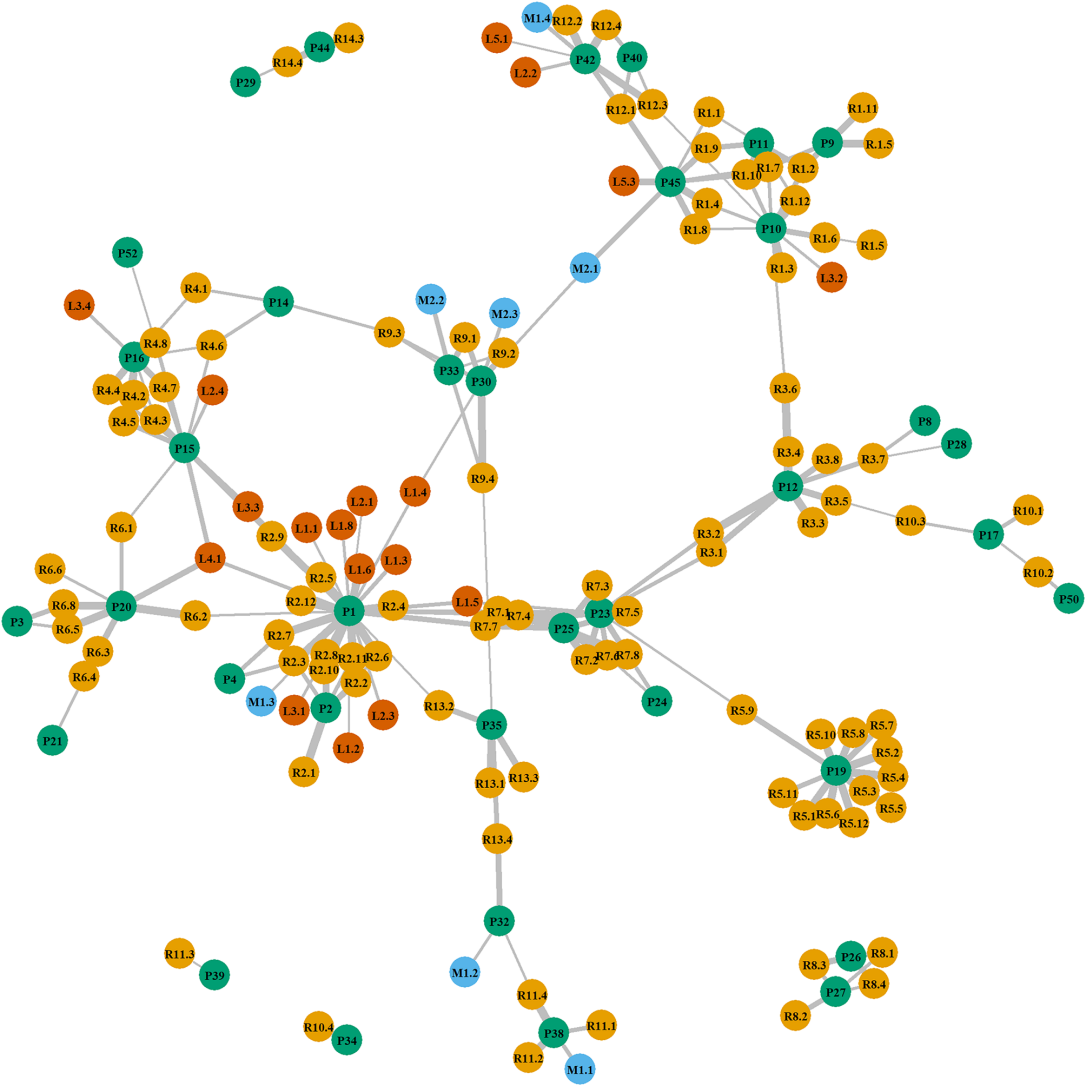
Match network from dust samples. Location and sample name: orange R_n_ = office, blue M_n_ = meeting room, red L_n_ = laboratory; the value after the decimal indicates the sample name from a specific location. The green nodes report the participants identified (P_n_). The network includes only matches that provided an LR > 100. The distance between nodes is inversely proportional to log_10_LR, the thickness of a node is directly proportional to log_10_LR: i.e. the shorter and thicker the edge, the higher log_10_LR value. The corresponding log_10_LR values for each sample are displayed in Supplementary Data 4.

Results of pairwise comparisons of dust samples are facilitated by the dnamatch2 program^38^. The results are represented by a match network (Fig. 6); the corresponding log_10_LR values for each sample represented in Fig. 6 are reported in Supplementary Data 4. The network is useful for investigative purposes because it can show which occupants contributed to which samples, thereby giving a valuable overview of potential movements of individuals. For example, it is notable that individual P1 (identified as high shedder from a previous internal study) appears in multiple locations, whereas others, such as P34 are observed once only. Participant P1 was detected from two air samples and from 11 of 12 dust samples recovered from the participant’s own office (R2). This individual was also identified in one air and one dust sample collected from a meeting room (M1), in seven dust samples collected from the washing room (L1) and in five samples recovered from DNA casework laboratories (L2-L4). In addition, P1 was detected as a non-occupant in five samples collected from different offices (Fig. 6). Three of these were from one office that the participant had been occupying until four months previous to sample collection.

Three other dust samples matched with non-occupants who declared that they had been working in an office in the past, ranging from one month to up to two years prior to sampling. Two of these samples were collected from the top of mirror frames, a hollow surface not typically cleaned, hence a perfect undisturbed place for indoor dust to accumulate and persist for long period of time.

DNA from another participant (P25), who had also been identified as high shedder from a previous study, was detected in DNA mixtures obtained from two air samples collected from this participant’s own office (R7) with log_10_LR values of 12 and 10, respectively; at the time of sampling, the individual had been absent from the office for 20–30 days. 

Overall, in offices with multiple occupants, it was observed that certain individuals were detected more frequently than others.

## Discussion

Previous research has shown that human DNA can be successfully collected and isolated from dust samples^19^. The current study, utilising improved DNA technology, reaffirms these findings. However, dust samples consistently yielded complex DNA mixtures, while air samples produced both partial single profiles and DNA mixtures. Dust is a reservoir of human skin cells and biological material, which can originate from current and past occupants of a specific location. DNA accumulated in dust can persist for a long time, from a few months up to a few years, depending on environmental factors such as light, heat, moisture, and cleaning agents, which may cause degradation^19,39–41^. On the other hand, DNA present in the air is more likely to represent individuals who recently occupied a room. The distribution of likelihood ratio (LR) values for different references varied among air and dust samples collected from different locations. Offices yielded the highest LR values, followed by meeting rooms and laboratories, influenced by the usage patterns. Individuals spending more time in an area, such as offices, are likely to disperse higher amounts of cells and DNA compared to those spending less time, as in meeting rooms. Areas where individuals spend more time during the day (i.e. offices) tend to have higher DNA transfer rates. Reither et al.^27^ found that the total amount of DNA recovered from household floors increased as residents spent more time at home. Puliatti et al.^17^ found that the amount of DNA transferred by individuals to non-touched surfaces near their workstation was time dependent. Cells and DNA can indeed be shed in the air directly by an individual from speaking, coughing or sneezing^12,13^, but can also be transferred indirectly from skin, clothes and personal items^6,9,15,16,42,43^. Cleaning habits also affect levels of DNA found on surfaces and in the air. Taylor et al.^44^ showed that sensitive areas, such as laboratories, had fewer inclusionary LR values compared to non-sensitive areas such as offices. In this study, the cleaning regime in sensitive areas helped to reduce detection of employees’ DNA; however some high LR values matching laboratory’s staff members were still found. We showed that detectable levels of human DNA are present in the dust analyzed from isolated areas of laboratories such as door ledges, which if disturbed, could be a potential source of contamination of casework material.

Two air samples collected from laboratories (washing room and DNA extraction laboratory), resulted in DNA mixtures with alleles at all 23 loci, but when analyzed using dnamatch2, did not provide matches with any of the known participants i.e. these profiles were of unknown origin. Remaining air samples collected from laboratories resulted in DNA profiles of poor quality (i.e. few or no alleles detected).

Success rates varied between the three environments. Offices had higher success rates compared to meeting rooms and laboratories. Non-occupants’ success rates were lower in dust samples from offices and laboratories, while higher rates were found for air samples collected 16 h after occupancy. The frequent air changes in the locations selected for the experiments of this study contributed to declining DNA recovery over time and may have limited detection to recent occupants. Ventilation can also transfer dust containing non-occupants DNA between rooms^19^. Opening doors can introduce external DNA from a corridor into a room; non-occupants DNA on surfaces may also disperse into the air through normal activity or mediated by investigator involvement. Moreover, the different air exchange rates in different rooms may have influenced the levels of DNA that could be recovered from the air. Future studies should consider ventilation levels and their types. The experiments described in this study were performed in a “clean” office environment for practical reasons: it was important to have the availability of a reference database from occupants of the premises. Further investigations into air and dust sampling should be performed in different indoor spaces (e.g. common households), since these are more likely to resemble a potential crime scene. DNA was found in restricted access areas of laboratories, suggesting the presence of individuals who were not regular occupants. This raises concerns in trace evidence examinations, as DNA can move between rooms in controlled environments. Undisturbed and inaccessible surfaces pose a lower contamination risk when collecting dust samples. However, DNA accumulates on undisturbed surfaces, not only from occupants but also from adjacent areas, despite occupants following strict cleaning protocols and wearing protective clothing. Disturbed dust becomes an aerosolized contamination risk, regardless of protection levels. Maintaining comprehensive staff elimination databases is vital, to include individuals working in offices and visitors, to detect the potential transfer of aerosol DNA within a facility^45,46^. 

The number of occupants in a location inversely affected the likelihood of detecting DNA from individual occupants. Additionally, the probability of identifying all occupants at least once depended on the total number of occupants in the location. To aid in determining the optimal number of dust samples needed to detect all inhabitants with a 95% probability, where sample size was taken into account, we developed a Bayesian statistical model. Our observations revealed that as the number of occupants increased, a greater number of samples was required to capture the majority of individuals. Although the model was solely tested on dust samples from offices, due to limited data for meeting rooms and laboratories, it serves as a suitable framework for future studies with more extensive datasets. Since occupants may traverse multiple locations (i.e. offices, meeting rooms and laboratories), a graphical model was used to map individuals to respective locations: this provides a demonstration of the utility of air/dust examination to aid criminal investigations where potential movement and past location of individuals is an important motivation. One individual (P1) could be detected in 32 air and dust samples from different offices, meeting rooms and laboratories. This individual was known from previous studies to be a high shedder. Shedder status may have an influence not only on the DNA deposited upon contact with items or surfaces^47–53^, but also on the DNA shed into the environment. The concept of shedder status is related to an individual’s natural propensity to shed DNA into the surrounding environment and may be influenced by factors such as personal and hygiene habits, behaviors and activities performed^17,54^.

DNA present in dust samples can serve as an historical record of individuals who were present many years ago, as DNA degrades slowly in dry conditions. Touch DNA left on surfaces, such as fingerprints, can be easily removed, indicating the potential for aerosolization^55,56^. Indoor dust undergoes constant re-aerosolization, allowing skin flakes and attached DNA to circulate in the air^57^. In a specific example, an individual (P25) who was absent from office R7 for 20–30 days appeared as a contributor in two samples collected from that office immediately after it was occupied by two colleagues. This individual, known to be a high shedder, may have had some of his DNA present on his desk or personal items, which could have been aerosolized by his colleagues during their regular activities.

The forensic laboratory has no a priori knowledge about the origin of DNA that may become evidence in a case. A contribution to a crime-sample may come from the result of a crime event itself or it may be present beforehand as environmental background DNA (from unknown persons) or as prevalent DNA (from known persons). To evaluate evidence, interpretation is carried out using the “hierarchy of propositions” framework^58,59^ which separates propositions of identity (sub-source) from the activity which led to the deposition of evidential material (activity level propositions). To evaluate DNA profiling evidence at the activity level, forensic scientists use a probabilistic framework that already takes account of risks of environmental background and prevalent DNA transfer, including secondary transfer, using Bayesian networks^60,61^ and this minimises risks of potential misreporting^62^.

Recently, Whitmore et al.^63^ described recovery of human DNA from water, air and sand. They used untargeted shotgun-sequencing and were able to detect genetic information. They observed that the amount of DNA detected was dependent on the distance from nearest human residence. Humans leave behind traces of their DNA at any location they occupy and this DNA can transfer to the nearby surroundings. With the rapid advances in DNA technologies, it is now possible to detect single fragments of human DNA in environmental samples. From a forensic perspective this gives new opportunities to detect DNA from occupants of a crime scene, but before adapting any new method it is important to understand the limitations, especially in the context of transfer, prevalence, persistence and recovery. Hence, more research is needed before this method can be adopted to criminal investigations.

In forensic DNA sampling there is always the possibility to collect DNA from persons not involved in a crime. In contrast to the whole genome sequencing approach utilized by environmental scientists, the traditional forensic DNA profiling does not obtain information about genes or genetic variants, it will only provide information about the identity of the donor if compared with his/her reference profile. The method proposed here does not offer any genetic information that exceeds that already available from traditional forensic samples, and furthermore is compatible with national DNA databases. The method is “investigative” rather than “evaluative”. Consistent with current practice, this means that the purpose is to provide a candidate list of references to investigators, where this list may contain both false positives and false negative results. Much more information is needed before an evaluative report can be prepared and the findings can be considered fit for testimony. Regardless, when new methods or genetic markers are applied in forensic genetics, it is always necessary to properly consider the ethical implications that may arise. 

In conclusion, although environmental DNA in forensic genetics has been known about for more than 20 years, the focus has been on contamination issues and associated risk management that includes use of restricted laboratories and protective clothing, along with well-established probabilistic Bayesian frameworks to interpret evidence. Here the focus has shifted in order to show how environmental DNA can be beneficially used in criminal investigations.

Novel methods were developed to collect and isolate human DNA from the air and dust. It was shown that air samples can provide STR genotypes of recent room occupants, while DNA from dust samples can identify individuals who occupied the room over longer periods. The study also revealed detectable levels of DNA in air and dust samples from forensic laboratories, improving our understanding of potential contamination sources.

The Bayesian models introduced can characterize DNA transfer in indoor dust and be used to estimate the number of samples needed to detect all occupants. The information obtained from air and dust samples may be used to search national DNA databases for intelligence purposes, identifying individuals who may have been present in the premises at recent and more distant times.

## Methods

### Ethical declaration

This study was approved by the data protection officer (DPO) at Oslo University Hospital (reference: 20/20155). Written informed consent was obtained from all participants. All experiments were performed in accordance with the relevant guidelines and regulations.

### Sample collection

A total of 184 samples were collected and analyzed for this study, consisting of 40 air samples and 144 dust samples. The study was conducted in the premises of the Forensic Genetics Unit at Oslo University Hospital. Out of 64 regular employees, 55 consented to take part in the study. Fourteen offices (with 1–7 occupants), two meeting rooms and five laboratories, connected by corridors, were used as the test site.

The first experiment examined dust and air DNA from the 14 different offices. The office environment is well defined, since the occupants are known, along with duration of occupancy. In the second experiment, similar studies were carried out with respect to two communal meeting rooms; here conditions were less well defined: the potential number of occupants is much higher than per office, but the frequency and duration of occupancy was much lower per individual. The third experiment was an examination of laboratories where access is restricted and protective clothing is worn to minimize risks of cross contamination.

### Air samples

This study was conducted in hospital premises which is provided with an efficient ventilation system that enables high air exchange rates. A double filtering system (filter classes F7 and F9) is installed to introduce outdoor air into the premises. The total air volume of offices is completely changed around 5 times per hour, depending on the room dimensions; in meeting rooms, air is completely changed 8 times per hour while air in laboratories is changed 10 times per hours.

A total of 41 air samples were collected. Seventeen air samples were collected from offices directly after occupancy (0 h), eight samples were collected c.16 h after occupancy (not consecutive to the 0 h samples), two samples were collected three days after occupancy and one 15 days after occupancy. Seven air samples were collected from the two meeting rooms and six samples were collected from five laboratories directly after occupancy. In the intervening period, rooms were left undisturbed. One air sample collected from a meeting room was excluded from the dataset, as there was evidence of contamination that occurred from the researcher that collected the sample (Supplementary Data 1). Participants were asked complete a questionnaire to gather information about their activity in their office during the sampling day; specifically, each participant declared how many hours they spent in the office, who was the last person to leave the office and if the office had been visited by other individuals. Samples were collected using the AirPrep™ ACD220 electret filter air sampler (Innovaprep^®^), a device provided with a 360° aerosol inlet, working with a 52 mm electret filter^64^. All rooms were unoccupied during sample collection. To set up the equipment, a single researcher entered the room with full personal protective equipment (lab coat, hairnet, face mask and gloves) and remained in the room just for the time necessary to set up the air sampler. The reference profile of the researcher that collected and processed the samples was compared to the DNA profiles obtained from all air samples to detect adventitious contaminations. The air sampler was run continuously for 2 h at a maximum flow rate of 200 LPM, positioned on a standard off-camera tripod at a distance of 110 cm from the floor. The sampler was located in the middle of the room, as far away from any furniture (e.g. chairs, desks) as possible, to avoid the collection of particles from nearby sources.

After collecting the sample, the air filter was secured in a sterile, pre-labeled, zip-lock bag and stored at room temperature until further processing. The whole sampler surface was cleaned before each sample collection using 70% ethanol and subsequently UV irradiated for 15 min. The external sampler surface and the tripod were further cleaned with a 0.1% hypochlorite solution to avoid cross-contamination. Two control samples were collected from the sampler’s internal surface that comes in contact with the air filter and two from the sampler’s lid, using moistened cotton swabs, after two different cleaning procedures, to evaluate their efficacy. In each test carried out, all four samples provided negative results (no CT) when quantified with PowerQuant^®^ System (Promega) on the 7500 RealTime PCR System (Applied Biosystems), showing that no detectable biological material was present on the sampler surfaces after the cleaning process.

### Dust samples

A total of 144 dust samples were collected in parallel to air samples (Supplementary Data 1). Dust samples were collected on the same day as the corresponding air samples, immediately after the air sample collection was concluded. Samples were taken using moistened cotton swabs from different dusty undisturbed surfaces that were not subject to regular cleaning, including door ledges, radiators, electric fittings and window sills. Samples were collected by swabbing a small area with light pressure, for about 10 s. Each cotton swab was placed into a labeled evidence bag and stored at room temperature until further processing.

### Sample processing

Samples were processed in a “DNA-free” investigation room. To avoid contamination the researcher handling the samples wore personal protective equipment during sample processing. Gloves were changed between each sample. Air samples were processed separately from dust, and samples from different rooms were processed on clean bench surfaces wiped with 0.1% hypochlorite solution, and covered with new bench paper. Each filter (air sample) was extracted from its zip-lock bag and the filter membrane was removed from its plastic shell using sterile scissors and tweezers. The filter membrane was cut in half and each half was cut into small pieces so that they could fit into two 2 ml extraction tubes. Each cotton swab (dust sample) was extracted from its sampling bag; the cotton swab tip was cut and placed into a 2 ml extraction tube. Samples were stored at room temperature until DNA extraction.

### DNA extraction

The DNA extraction from the air filters was performed following a modified QIAamp^®^ DNA mini kit (Qiagen) procedure. The protocol was adapted from Clare et al.^65^ and Duhaime and Cable^66^. The extraction of one air sample was carried out using two 2 ml extraction tubes, each containing half-filter cut into small pieces. Cell lysis was performed by submerging all filter pieces into 450 µl of ATL and 500 µl of AL lysis buffers and 50 µl of Proteinase K and incubating the samples at 56 °C, shaking them at 650 rpm for 1 h. The samples were further shaken at maximum speed for 10 min, before centrifugation at 17.000 *g* for 30 s. The filter pieces were subsequently transferred using sterile tweezers, along with the sample lysate, into QIAshredder columns and centrifuged at 17.000 *g* for 15 s. The transfer was performed in multiple steps, until all filter pieces and lysate had been moved to the columns. After discarding the QIAshredder columns with the retained dry filter pieces, 500 µl of 100% ethanol was added to the solution and mixed by vortexing. Following the manufacturer’s protocol, the mixture of lysate and ethanol was transferred onto the QIAamp DNA mini spin columns and centrifuged at 17.000 *g* for 15 s. The columns were then washed with 500 µl of AW1 wash buffer and centrifuged for 15 s at 17.000 *g*, and further washed with AW2 wash buffer and centrifuged at 12,000 *g* for 3 min. A second centrifugation step was done at full speed for 1 min. The sample was eluted using 30 µl of AE elution buffer, preheated to 70 °C after an incubation of 5 min and was centrifuged for 1 min at ≥ 8000 *g*. The elution step was repeated by passing the first eluate through the column a second time. Finally, since one filter was processed in two tubes, the eluates from the two tubes were pooled, resulting in 60 µl of extract. Negative extraction controls were extracted along with the samples. Dust samples were extracted following the same protocol as described for air samples, but processing the cotton swab tip in a single tube, resulting in 30 µl of extract.

### DNA analysis

Samples were quantified using the PowerQuant^®^ System (Promega) on the 7500 RealTime PCR System (Applied Biosystems) and amplified using PowerPlex^®^ Fusion 6C (Promega) kit with 1.0 ng template DNA input in a final reaction volume of 25 µl. Applied Biosystem^®^ Veriti 96-Well Thermal Cycler (ThermoFisher) with a 29 PCR cycles program was used for the amplification. Negative and positive controls were run with the samples. Capillary electrophoresis was performed on the Applied Biosysetm^®^ 3500xL Genetic Analyzer (ThermoFisher) with an injection time of 24 s and a voltage of 1.2 kV.

### Data analysis

The data were analyzed using GeneMapper ID-X v.1.6 (Life Technologies) with an analytical threshold (AT) set to 50 RFU for air samples and to 100 RFU for dust samples. Technical artefacts were filtered from the DNA profiles, according to internal guidelines. DNA profiles obtained from air samples were evaluated in relation to the use and length of time since occupancy of the room from where samples were collected. DNA profiles obtained from dust samples were evaluated in relation to the use of the room and the number of room occupants. A comparative analysis of the results obtained from air and dust samples was also conducted.

### Calculation of the likelihood ratio

A likelihood ratio framework was applied to assign the value of the evidence conditioned on a given person of interest (*PoI*), e.g., the location occupant. An LR was calculated for each matching reference sample from the database as the *PoI* in turn (Eq. (1)).

Likelihood ratio (LR) values given by Eq. (1) were calculated from evidence profile *E*, given two alternative propositions (*H*_*1*_) and (*H*_*2*_), where ${H}_{1}$ is: “$PoI$ and $L-1$ unrelated unknowns are contributors”, versus ${H}_{2}$: “$L$ unrelated unknowns are contributors”. A reference genotype database *R* of the consenting 55 occupants of the building participating in the study was compiled. Under ${H}_{1}$ the reference profile *PoI* is conditioned, which is an individual from the reference database $R$. Every DNA profile retrieved was compared against each reference in the database (POI_1:55_) using dnamatch2^38^ (http://www.euroformix.com/dnamatch2). This open-source software is a fast, large-scale DNA database search algorithm based on EuroForMix (http://www.euroformix.com/, version 4.0.3 applied).

The analysis enabled identification of “matches” with genotypes of the 55 known occupants of the building and to calculate the corresponding LR values, the number of contributors (NOC) and mixture proportions (*M*_*x*_). The following settings for dnamatch2 were used for the analyses: score thresholds: matching allele counting (MAC) = 0.3, qualitative and quantitative LR = 1; model set-up: drop-in probability = 0.05, Lambda parameter = 0.01, min freq = 0.001; prefilter thresholds: analytical threshold (AT) = 50 RFU, stutter rate threshold = 0.1, major extraction rate threshold = 0.6, minimum loci requirement (Evid and Maj) = 3; the maximum number of contributor was set to 4. Stutter models not applied.

To minimize adventitious or false hits with the database, a match of the evidence with a reference sample was defined as a likelihood ratio (LR) greater than 100. The reference database used in our study was relatively small, however there is no restriction placed upon its size: indeed, massive intelligence databases of several million individuals are amenable. In practice, comparisons with large intelligence databases size N, will require high LRs in order to reduce the number of false positive matches. For example, if the database size is 1 m, and the LR recovered is 10,000, from the Turing expectation c. 100 adventitious matches may be observed in such a database (Pm = N/LR)^35–37^. Smaller databases (N = 55), such as that used here will result in much lower adventitious match rates. Therefore, for this experiment we used an arbitrary LR = 100 as a reasonable threshold to distinguish true positives from false negatives; but the optimum threshold used depends upon the size of the intelligence database used.

The final list of candidate matches and relative LR values was further evaluated with respect to the information available regarding each sample, such as the identity of the individual(s) occupying a specific room and the information gathered through the activity questionnaire (for air samples). This enabled us to establish the number of known occupants, and known non-occupants detected for each sample collected. This study focused on known individuals (i.e. the participants of the study); unknown contributors (i.e. individuals not present in the reference database) were not considered. LR values were compared between air and dust samples collected from the same locations.

### Outline of the model

The following factors and variables were recorded:

1. Location: $z=1,\dots Z$. Where locations are offices (R), meeting rooms (M) and laboratories (L).
2. ${K}_{z}$: Number of unique, known occupants in the location.
3. *k*_*z*_: A specific occupant from location *z*.
4. ${S}_{z}:$ Number of samples taken from location *z* (over a period of days).
5. ${N}_{z}$: Number of samples from location *z* where no known occupants were positively identified.
6. ${NC}_{z}$: Number of samples from location *z* where no known non-occupants were positively identified.
7. *X*_*k,z*_: Number of samples from location *z* with positive identification of occupant *k*_*z*_.
8. *φ*_*1*_: Success rate for occupants, defined as the proportion of samples where one or more occupants were detected (Eq. (4)).
9. *φ*_*2*_: Success rate for non-occupants, defined as the proportion of samples where one or more known non-occupants were detected (Eq. (5)).
  4 $${\it {\varphi }_{1}}=1-\frac{\sum_{z}{N}_{z}}{\sum_{z}{S}_{z}}$$
  5 $${\it {\varphi }_{2}}=1-\frac{\sum_{z}{NC}_{z}}{\sum_{z}{S}_{z}}$$

### Statistical analysis

Wilcoxon and Fisher exact two-tailed tests were used to determine if the expectations were the same in different groups with a 5% significance level. RStudio 2022.07.2 was used to perform the statistical analyses and the packages ggplot2 v. 3.4.0 and igraph 1.3.5 were used to generate the figures presented in this manuscript.

### Supplementary Information

Dataset S1.

Dataset S2.

Dataset S3.

Dataset S4.

Supplementary Information.

## Data Availability

The data and programs used to carry out the logistic regression and Bayesian analysis in this study are available at: https://github.com/peterdgill/DustandAir.
