# Supplementary material for: The invisible witness: air and dust as DNA evidence of human occupancy in indoor premises
Source: Sci Rep. 2023 Nov 4;13:19059. doi: 10.1038/s41598-023-46151-7 (PMC10625553; doi:10.1038/s41598-023-46151-7)
Supplement: Supplementary file 5 — Supplementary Information. [file 41598_2023_46151_MOESM5_ESM.docx]

**Supplementary information**

**The invisible witness: air and dust as DNA evidence of human occupancy in indoor premises**

Chiara Fantinato^a,b,*^, Ane Elida Fonneløp^a,c^, Øyvind Bleka^a^, Magnus Dehli Vigeland^a^, Peter Gill^a,b^

*a. Department of Forensic Sciences, Oslo University Hospital, Oslo, Norway
b. Department of Forensic Medicine, Institute of Clinical Medicine, University of Oslo, Oslo, Norway
c. Centre for Ecological and Evolutionary Synthesis (CEES), Department of Biosciences, University of Oslo, Oslo, Norway*

***Corresponding author at: Department of Forensic Sciences, Oslo University Hospital, Oslo, Norway. *E-mail address:* [chifan@ous-hf.no](mailto:chifan@ous-hf.no) (C. Fantinato)

**This document contains Supplementary Tables 1-6 and Supplementary Figures 1-13.**

1. **DNA recovery from dust**

**1.1. Meeting rooms**

A total of 24 dust samples were collected from two meeting rooms and all samples provided DNA results; however, 17 samples did not provide any match with LR>100, (Supplementary Table 1).

**Supplementary Table 1: Data from dust samples collected from meeting rooms.***K_z_:* number of unique known occupants in location *z;* No. untyped occupants: number of known occupants not participating in the study whose reference profile was not included in the reference database. $S_{z}$: total number of samples collected and analyzed from each location. *N_z_*: number of samples from location *z* where no known occupants were positively identified, including samples with negative results (no DNA recovered/no matches with LR>100). P1,...,P4: number of times a known unique occupant is detected per location (z).

| Location (*z*) | $\boldsymbol{K}_{\boldsymbol{z}}$ | No. untyped occupants | $\boldsymbol{S}_{\boldsymbol{z}}$ | $\boldsymbol{N}_{\boldsymbol{z}}$ | P1 | P2 | P3 | P4 | No. unique occupants detected |
| --- | --- | --- | --- | --- | --- | --- | --- | --- | --- |
| M1 | 64 | 9 | 12 | 8 | 1 | 1 | 1 | 1 | 4 |
| M2 | 64 | 9 | 12 | 9 | 2 | 1 | 1 | 0 | 3 |
| Totals: | **64** | **9** | **24** | **17** |  |  |  |  | **7** |

**1.2. Laboratories**

Twenty-four dust samples were collected from three different types of laboratory facilities. The first laboratory, named in Table 2 as L1, is a washing room where tools used for casework sample recovery and analysis (e.g. scissors, tweezers) are washed. Laboratories L2-L4 are DNA casework laboratories: specifically, L2 is a DNA extraction laboratory, L3 is where samples are prepared for DNA amplification and L4 is where samples are prepared for DNA quantification. Finally, L5 is an investigation room where casework evidence material is processed. Since different individuals work in the three laboratory environments, to calculate success rates, the three areas were considered separately.

**1.2.1. Washing room**

A total of eight dust samples were collected from washing room L1, all of them providing DNA results. One sample did not give any match with an LR>100, (Supplementary Table 2). The room is accessed by 41 individuals, but only one of these was detected.

**1.2.2. DNA casework laboratories**

Twelve dust samples were collected from laboratories L2-L4 and 11 of these provided DNA results. However, two of those 11 samples gave matches that were below the match threshold of LR=100 , (Supplementary Table 2).

**1.2.3. Investigation room**

A total of four samples were collected from the investigation room L5. One of the samples did not provide DNA results and one did not provide any match with an LR>100, (Supplementary Table 2).

**Supplementary Table 2: Data from dust samples collected from laboratories.**L1: washing room, L2-L4: DNA casework laboratories, L5: investigation room; *K_z_:* number of unique known occupants in location *z;* No. untyped occupants: number of known occupants not participating in the study whose reference profile was not included in the reference database. $S_{z}$: total number of samples collected and analyzed from each location. *N_z_*: number of samples from location *z* where no known occupants were positively identified, including samples with negative results (no DNA recovered/no matches with LR>100). P1,...,P3: number of times a known unique occupant is detected per location (z).

| Location (z) | $\boldsymbol{K}_{\boldsymbol{z}}$ | No. untyped occupants | $\boldsymbol{S}_{\boldsymbol{z}}$ | $\boldsymbol{N}_{\boldsymbol{z}}$ | P1 | P2 | P3 | No. unique occupants detected | No. unique non-occupants detected |
| --- | --- | --- | --- | --- | --- | --- | --- | --- | --- |
| L1 | 41 | 5 | 8 | 1 | 7 | 0 | 0 | 1 | 2 |
| L2 | 15 | 1 | 4 | 1 | 2 | 1 | 0 | 2 | 1 |
| L3 | 15 | 1 | 4 | 1 | 2 | 1 | 1 | 3 | 1 |
| L4 | 15 | 1 | 4 | 3 | 1 | 1 | 1 | 3 | 0 |
| L5 | 24 | 6 | 4 | 2 | 1 | 1 | 0 | 2 | 0 |
| Totals: |  |  | **24** | **8** |  |  |  | **11** | **4** |

**2. DNA recovery from the air**

**2.1. Air samples collected from offices 16 h after occupancy**

Of the eight samples collected 16 h after occupancy, six provided DNA results and two of these did not give any match with an LR>100 (Supplementary Table 3). Only one over 20 known occupants was detected. Known non-occupants were detected in four samples.

**Supplementary Table 3: Data from air samples collected from offices at 16 h after occupancy.***K_z_:* number of unique known occupants in location *z;* No. untyped occupants: number of known occupants not participating in the study whose reference profile was not included in the reference database. $S_{z}$: total number of samples collected and analyzed from each location. *N_z_*: number of samples from location *z* where no known occupants were positively identified, including samples with negative results (no DNA recovered/no matches with LR>100). P1,...,P5: number of times a known unique occupant is detected per location (z).

| Location (*z*) | $\boldsymbol{K}_{\boldsymbol{z}}$ | No. untyped occupants | $\boldsymbol{S}_{\boldsymbol{z}}$ | $\boldsymbol{N}_{\boldsymbol{z}}$ | P1 | P2 | P3 | P4 | P5 | No. unique occupants detected | No. unique non- occupants detected |
| --- | --- | --- | --- | --- | --- | --- | --- | --- | --- | --- | --- |
| R1 | 4 | 0 | 1 | 1 | 0 | 0 | 0 | 0 | - | 0 | 1 |
| R2 | 5 | 0 | 1 | 1 | 0 | 0 | 0 | 0 | 0 | 0 | 0 |
| R3 | 1 | 0 | 2 | 0 | 2 | - | - | - | - | 1 | 3 |
| R5 | 1 | 0 | 1 | 1 | 0 | - | - | - | - | 0 | 3 |
| R6 | 3 | 0 | 1 | 1 | 0 | 0 | 0 | - | - | 0 | 0 |
| R11 | 2 | 0 | 1 | 1 | 0 | 0 | - | - | - | 0 | 0 |
| R12 | 4 | 1 | 1 | 1 | 0 | 0 | 0 | - | - | 0 | 0 |
| Totals: | **20** | **1** | **8** | **6** |  |  |  |  |  | **1** | **7** |

**2.1. Meeting rooms**

Six air samples collected from the two meeting rooms just after occupancy were analyzed and all provided DNA results; three of the samples however did not give any match with an LR>100, (Supplementary Table 4).

**Supplementary Table 4: Data from air samples collected from meeting rooms.***K_z_:* number of unique known occupants in location *z;* No. untyped occupants: number of known occupants not participating in the study whose reference profile was not included in the reference database. $S_{z}$: total number of samples collected and analyzed from each location. *N_z_*: number of samples from location *z* where no known occupants were positively identified, including samples with negative results (no DNA recovered/no matches with LR>100). P1,...,P3: number of times a known unique occupant is detected per location (z).

| Location (z) | $\boldsymbol{K}_{\boldsymbol{z}}$ | No. untyped occupants | $\boldsymbol{S}_{\boldsymbol{z}}$ | $\boldsymbol{N}_{\boldsymbol{z}}$ | P1 | P2 | P3 | No. unique occupants detected |
| --- | --- | --- | --- | --- | --- | --- | --- | --- |
| M1 | 64 | 9 | 4 | 1 | 1 | 1 | 1 | 3 |
| M2 | 64 | 9 | 2 | 2 | 0 | 0 | 0 | 0 |
| Totals: | **64** | **9** | **6** | **3** |  |  |  | **3** |

**2.2. Laboratories**

A total of six air samples were collected from laboratories L1-L5 just after occupancy, five of them provided DNA results (Supplementary Table 5). However, four of these samples did not give any match with an LR>100. Interestingly, two samples (one collected from a DNA extraction laboratory and one from the washing room) did not display any match even if they resulted in DNA mixtures with alleles detected at all 23 loci.

**Supplementary Table 5: Data from air samples collected from laboratories.**L1: washing room, L2-L4: DNA casework laboratories, L5: investigation room. *K_z_:* number of unique known occupants in location *z;* No. untyped occupants: number of known occupants not participating in the study whose reference profile was not included in the reference database. $S_{z}$: total number of samples collected and analyzed from each location. *N_z_*: number of samples from location *z* where no known occupants were positively identified, including samples with negative results (no DNA recovered/no matches with LR>100). P1: number of times a known unique occupant is detected per location (z).

| Location (z) | $\boldsymbol{K}_{\boldsymbol{z}}$ | No. untyped occupants | $\boldsymbol{S}_{\boldsymbol{z}}$ | $\boldsymbol{N}_{\boldsymbol{z}}$ | P1 | No. unique occupants detected |  | No. unique non-occupants detected | |
| --- | --- | --- | --- | --- | --- | --- | --- | --- | --- |
| L1 | 41 | 5 | 2 | 1 | 1 | 1 |  | 0 |  |
| L2 | 15 | 1 | 1 | 1 | 0 | 0 |  | 0 |  |
| L3 | 15 | 1 | 1 | 1 | 0 | 0 |  | 0 |  |
| L4 | 15 | 1 | 1 | 1 | 0 | 0 |  | 0 |  |
| L5 | 24 | 4 | 1 | 1 | 0 | 0 |  | 0 |  |
| Totals: |  |  | **6** | **5** |  | **1** |  | **0** |  |

**3. Probabilistic analysis of number of samples needed to give a Pr=0.95 and Pr=0.9 of detecting all occupants in an office**

The data displayed in Supplementary Table 6 are sorted by the number of occupants in the office and summarized accordingly. The final column in the table lists the range of results across all three methods for the 0.95 quantile, providing the observed minimum/maximum number of samples required to reach this value.
The number of samples needed to capture *K* occupants with 95% probability increases with *K*, hence for one occupant 2-5 samples are needed, rising to 13-21 for seven occupants.

**Supplementary** **Table 6: Dust in offices. Number of samples required to reach** $\boldsymbol{\phi}_{\mathbf{z}}\boldsymbol{=0.95}$ **and 0.9 using three methods of calculation.** The data are sorted according to the number of occupants and the final column gives minimum and maximum findings across the three methods across K values analyzed for $\phi_{z}=0.95$. *K_z_:* number of unique known occupants in location *z;* $S_{z}$: total number of samples collected and analyzed from each location.

| Location (z) | $\boldsymbol{K}_{\boldsymbol{z}}$ | $\boldsymbol{S}_{\boldsymbol{z}}$ | Exp 0.95 | Mode 0.95 | Median 0.95 | Exp 0.9 | Mode 0.9 | Median 0.9 | Range 0.95 |
| --- | --- | --- | --- | --- | --- | --- | --- | --- | --- |
| R3 | 1 | 8 | 2 | 2 | 2 | 2 | 2 | 2 |  |
| R5 | 1 | 12 | 2 | 2 | 2 | 2 | 2 | 2 | 2-5 |
| R14 | 1 | 4 | 5 | 5 | 5 | 4 | 4 | 4 |  |
| R9 | 2 | 4 | 4 | 3 | 4 | 3 | 3 | 3 |  |
| R11 | 2 | 4 | 7 | 7 | 7 | 5 | 6 | 6 | 3-7 |
| R13 | 2 | 4 | 6 | 7 | 7 | 5 | 5 | 5 |  |
| R6 | 3 | 8 | 14 | 18 | 17 | 11 | 14 | 14 |  |
| R7 | 3 | 8 | 7 | 8 | 8 | 6 | 6 | 6 | 8-18 |
| R8 | 3 | 4 | 9 | 11 | 10 | 7 | 9 | 8 |  |
| R1 | 4 | 12 | 7 | 7 | 8 | 6 | 6 | 6 |  |
| R4 | 4 | 8 | 13 | 17 | 15 | 11 | 13 | 12 | 7-17 |
| R12 | 4 | 4 | 11 | 13 | 14 | 9 | 11 | 11 |  |
| R2 | 5 | 12 | 21 | 28 | 28 | 17 | 23 | 23 | 21-28 |
| R10 | 7 | 4 | 13 | 16 | 21 | 11 | 13 | 17 | 13-21 |

**Supplementary Figures 1-13**


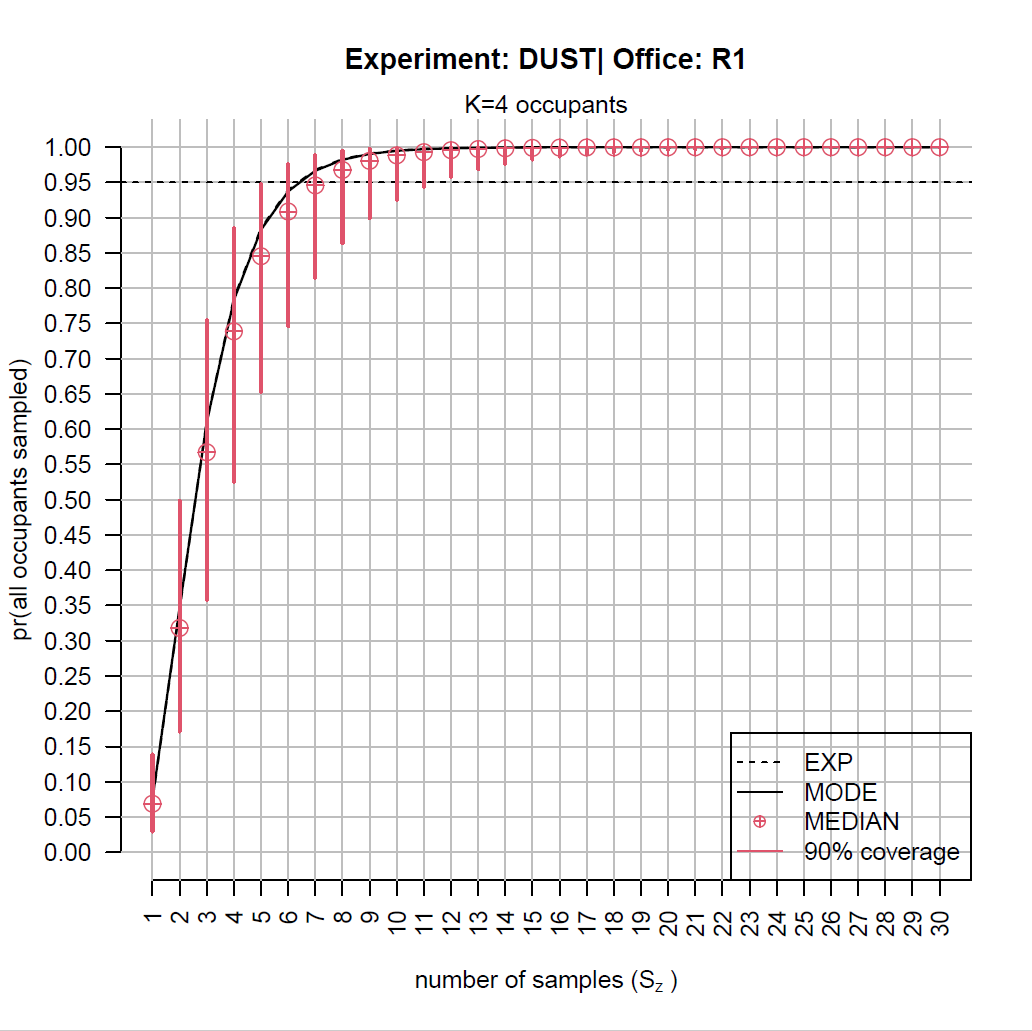


**Supplementary Fig. 1: Office R1, K=4: Plot of probability of detecting all k_z_ (**$\boldsymbol{\phi}_{\boldsymbol{z}}$**) at least once out of K occupants, in a set of samples, varying size (S_z_) using three different estimators.**


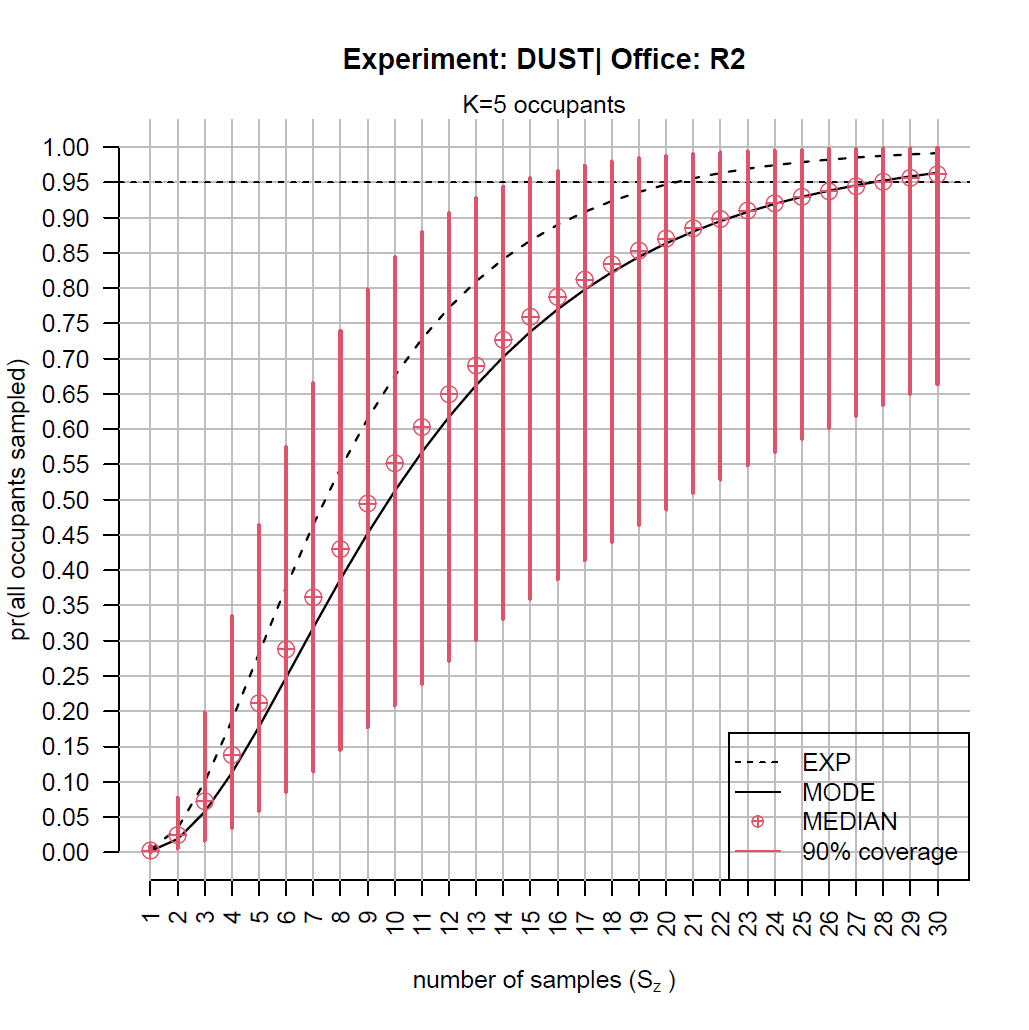


**Supplementary Fig. 2: Office R2, K=5: Plot of probability of detecting all k_z_ (**$\boldsymbol{\phi}_{\boldsymbol{z}}$**) at least once out of K occupants, in a set of samples, varying size (S_z_) using three different estimators.**


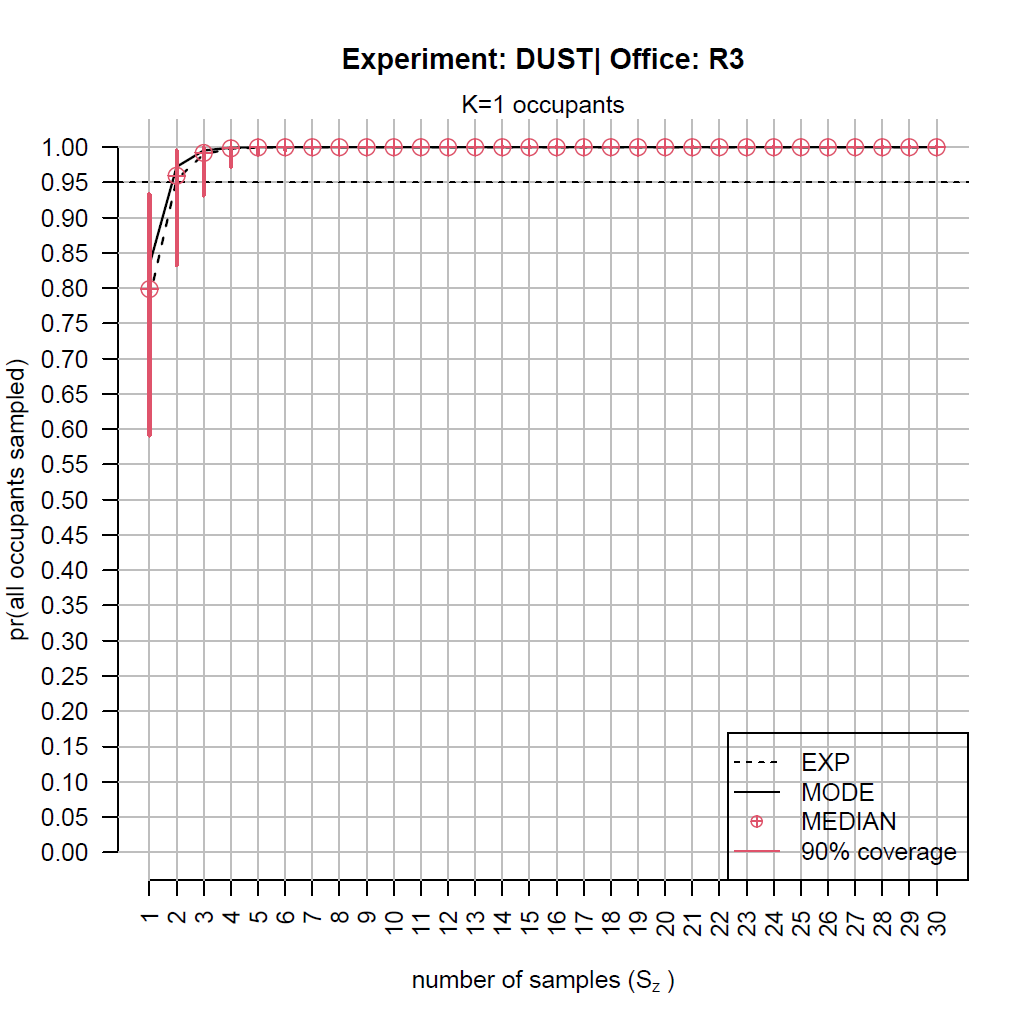


**Supplementary Fig. 3: Office R3, K=1: Plot of probability of detecting all k_z_ (**$\boldsymbol{\phi}_{\boldsymbol{z}}$**) at least once out of K occupants, in a set of samples, varying size (S_z_) using three different estimators.**


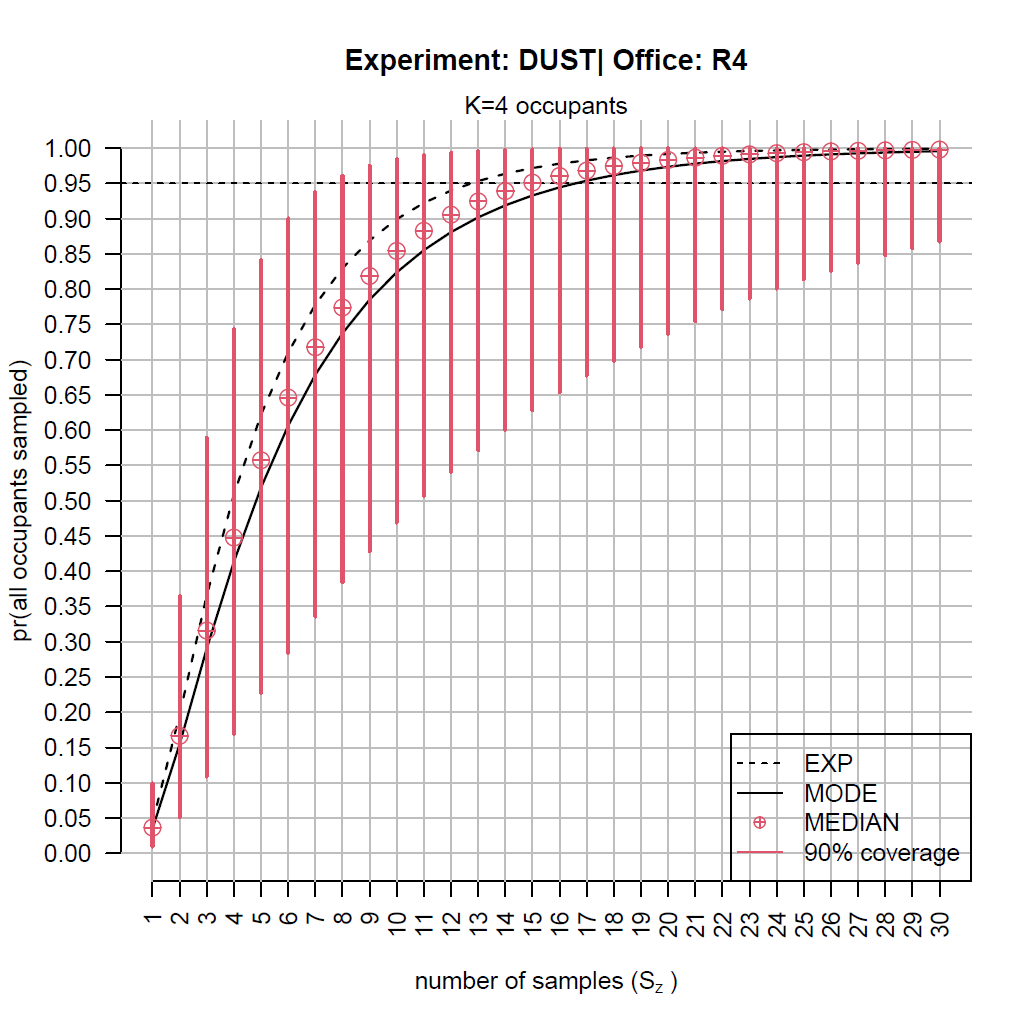


**Supplementary Fig. 4: Office R4, K=4: Plot of probability of detecting all k_z_ (**$\boldsymbol{\phi}_{\boldsymbol{z}}$**) at least once out of K occupants, in a set of samples, varying size (S_z_) using three different estimators.**


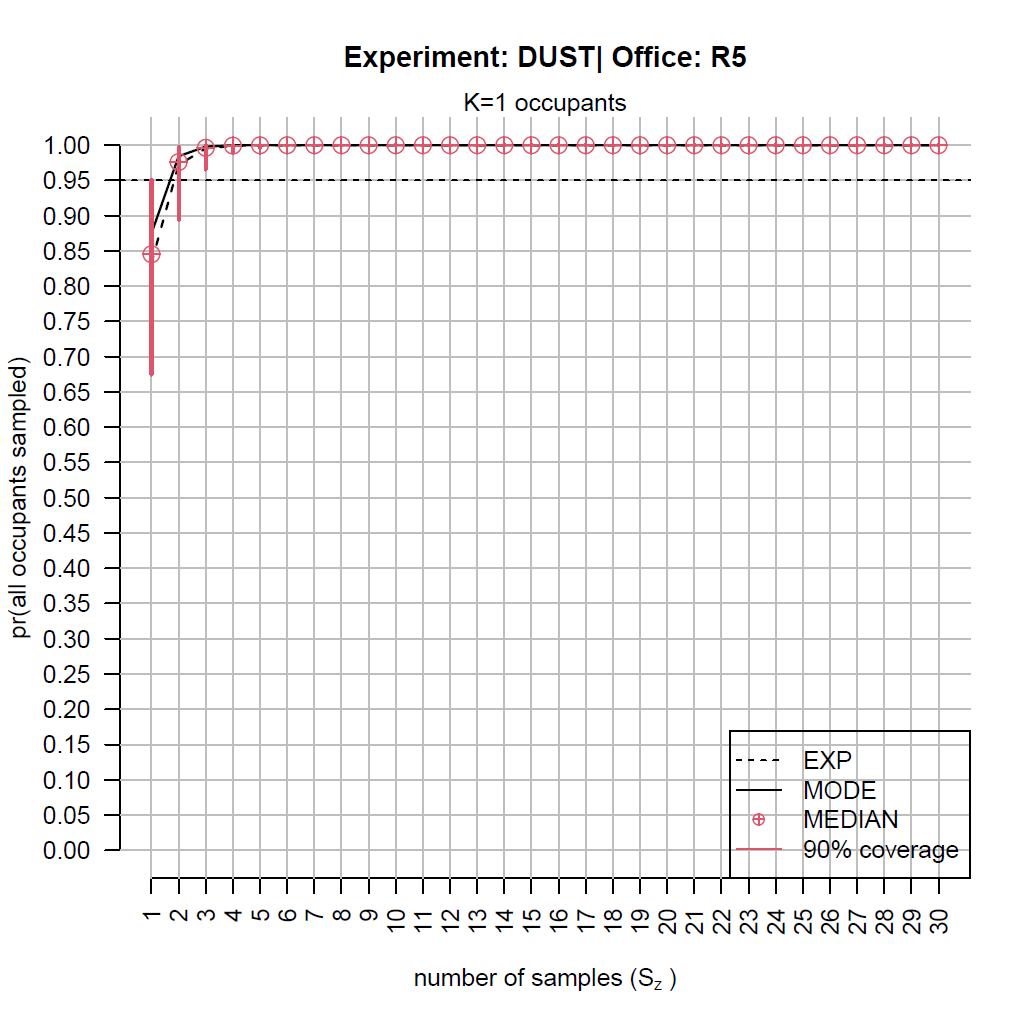


**Supplementary Fig. 5: Office R5, K=1: Plot of probability of detecting all k_z_ (**$\boldsymbol{\phi}_{\boldsymbol{z}}$**) at least once out of K occupants, in a set of samples, varying size (S_z_) using three different estimators.**


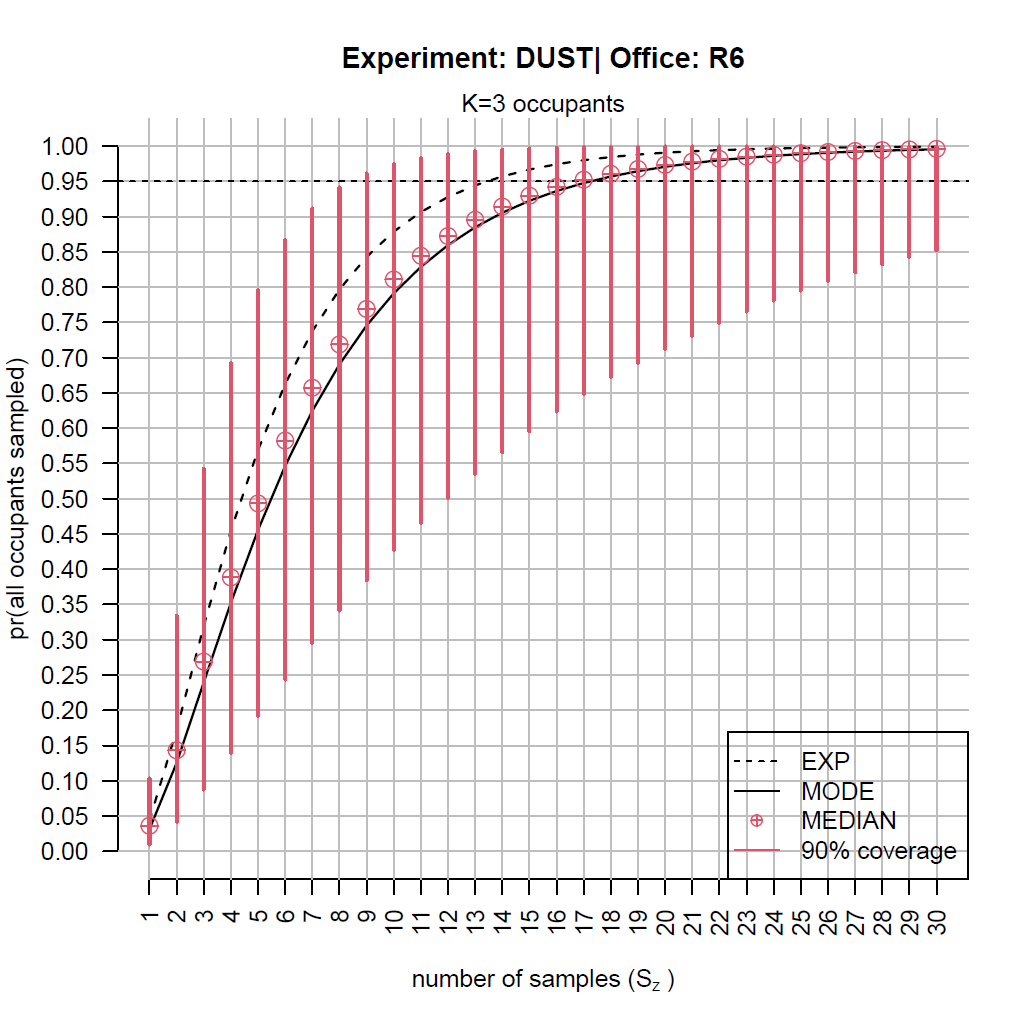


**Supplementary Fig. 6: Office R6, K=3: Plot of probability of detecting all k_z_ (**$\boldsymbol{\phi}_{\boldsymbol{z}}$**) at least once out of K occupants, in a set of samples, varying size (S_z_) using three different estimators.**


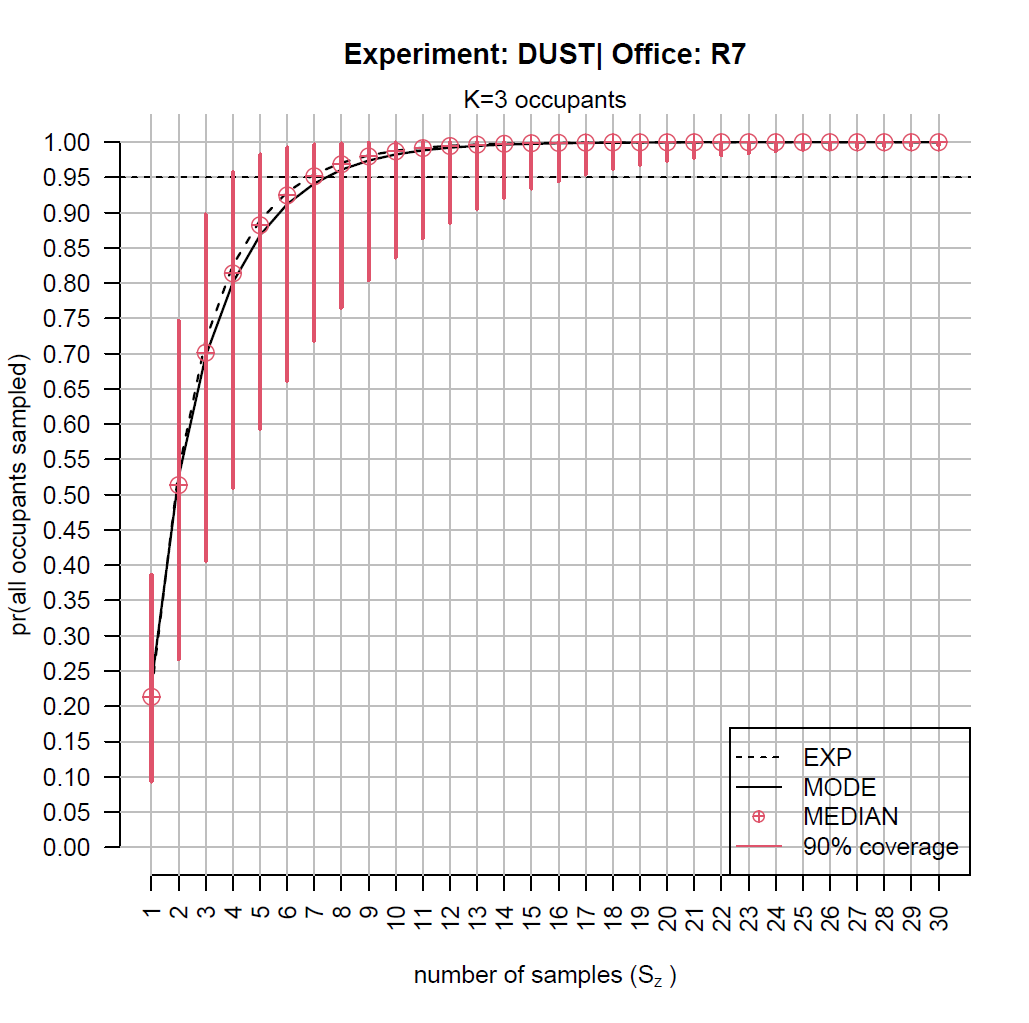


**Supplementary Fig. 7: Office R7, K=3: Plot of probability of detecting all k_z_ (**$\boldsymbol{\phi}_{\boldsymbol{z}}$**) at least once out of K occupants, in a set of samples, varying size (S_z_) using three different estimators.**


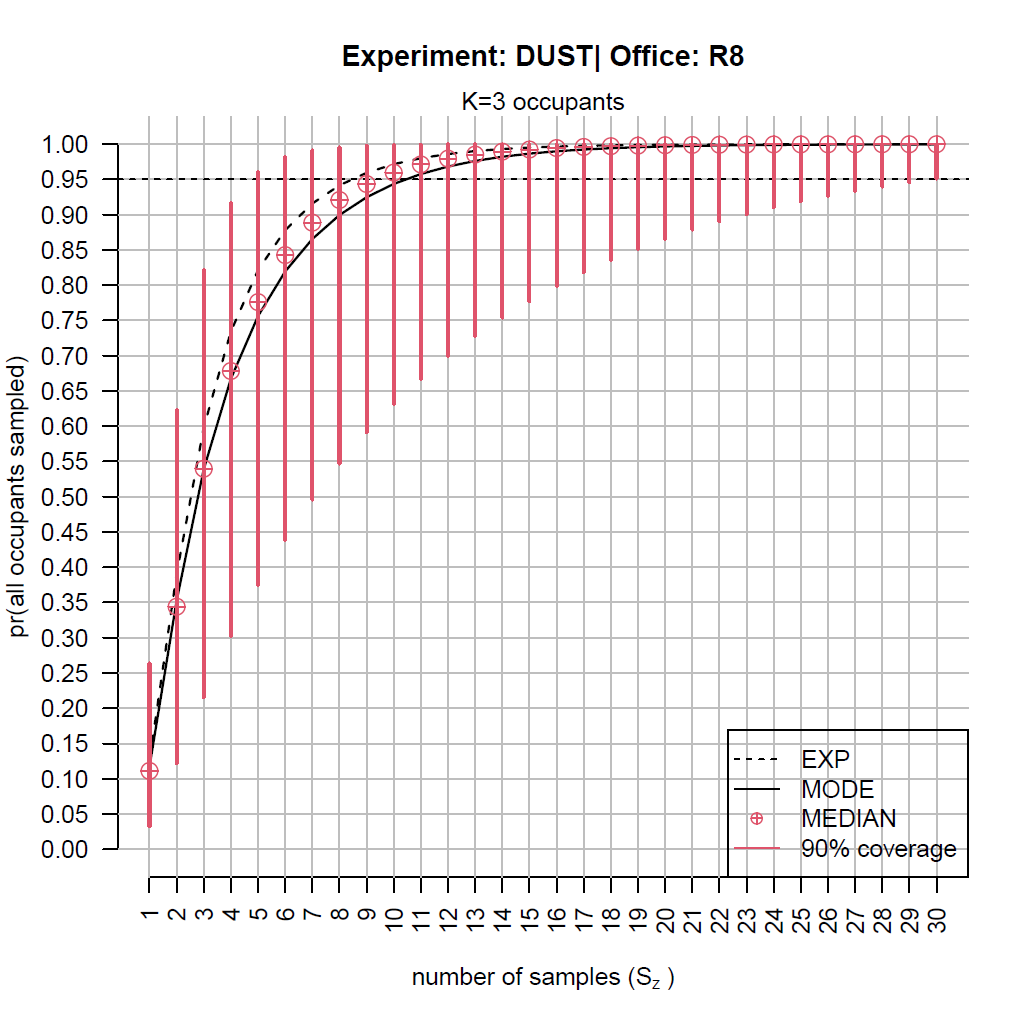


**Supplementary Fig. 8: Office R8, K=3: Plot of probability of detecting all k_z_ (**$\boldsymbol{\phi}_{\boldsymbol{z}}$**) at least once out of K occupants, in a set of samples, varying size (S_z_) using three different estimators.**


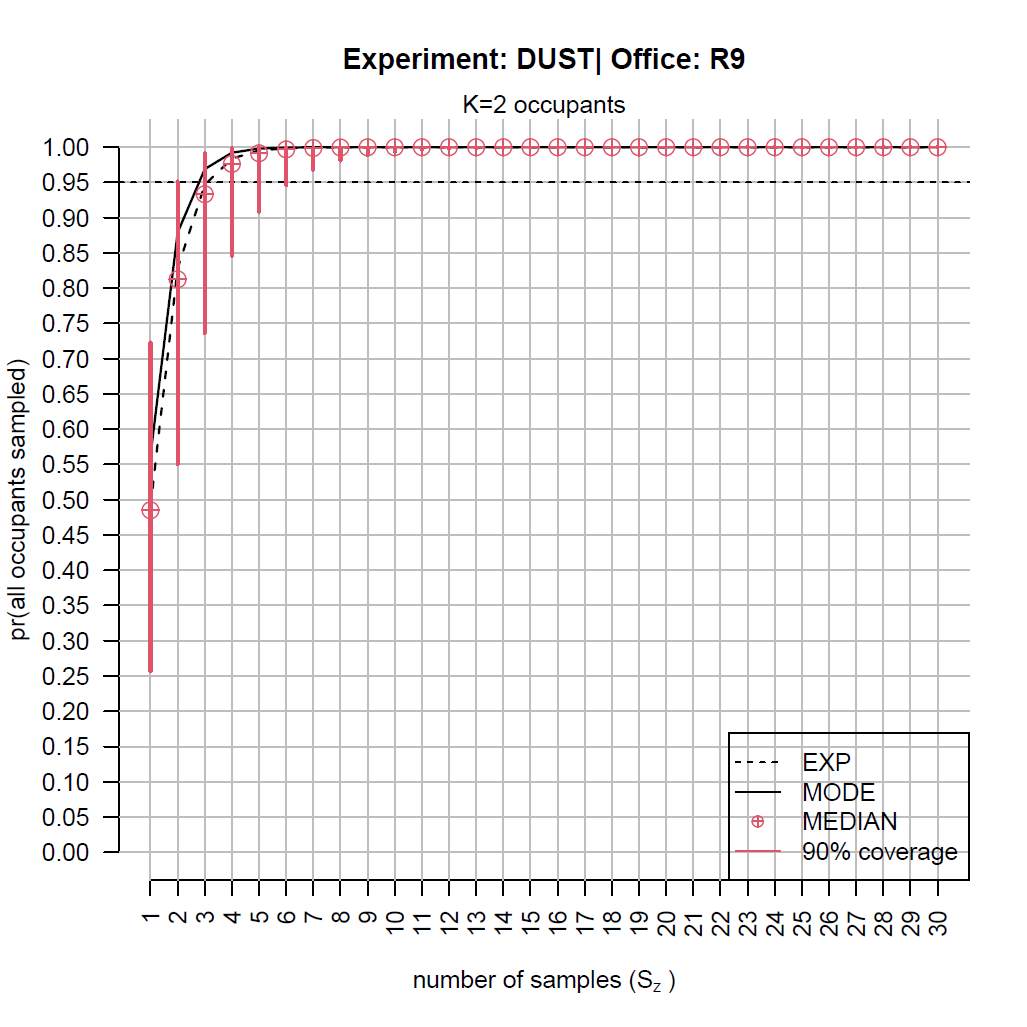


**Supplementary Fig. 9: Office R9, K=2: Plot of probability of detecting all k_z_ (**$\boldsymbol{\phi}_{\boldsymbol{z}}$**) at least once out of K occupants, in a set of samples, varying size (S_z_) using three different estimators.**


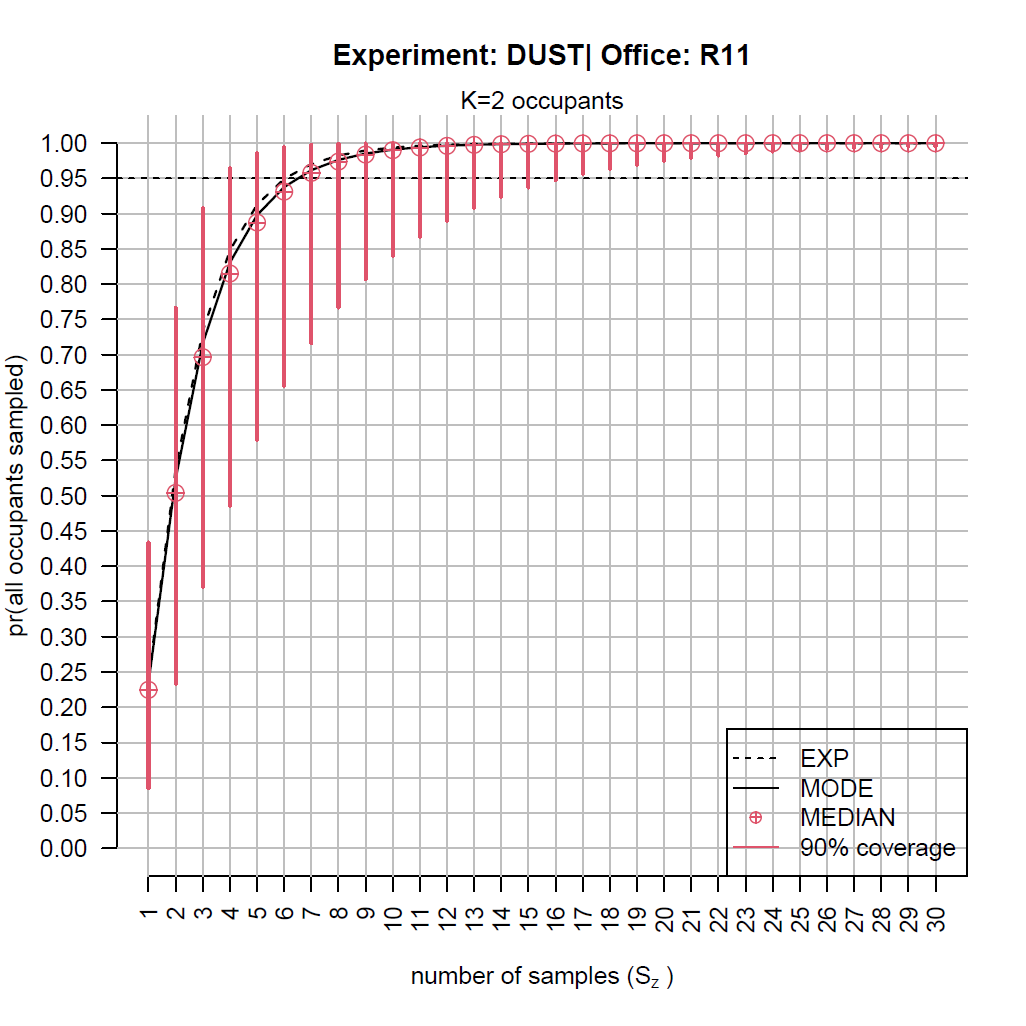


**Supplementary Fig. 10: Office R11, K=2: Plot of probability of detecting all k_z_ (**$\boldsymbol{\phi}_{\boldsymbol{z}}$**) at least once out of K occupants, in a set of samples, varying size (S_z_) using three different estimators.**


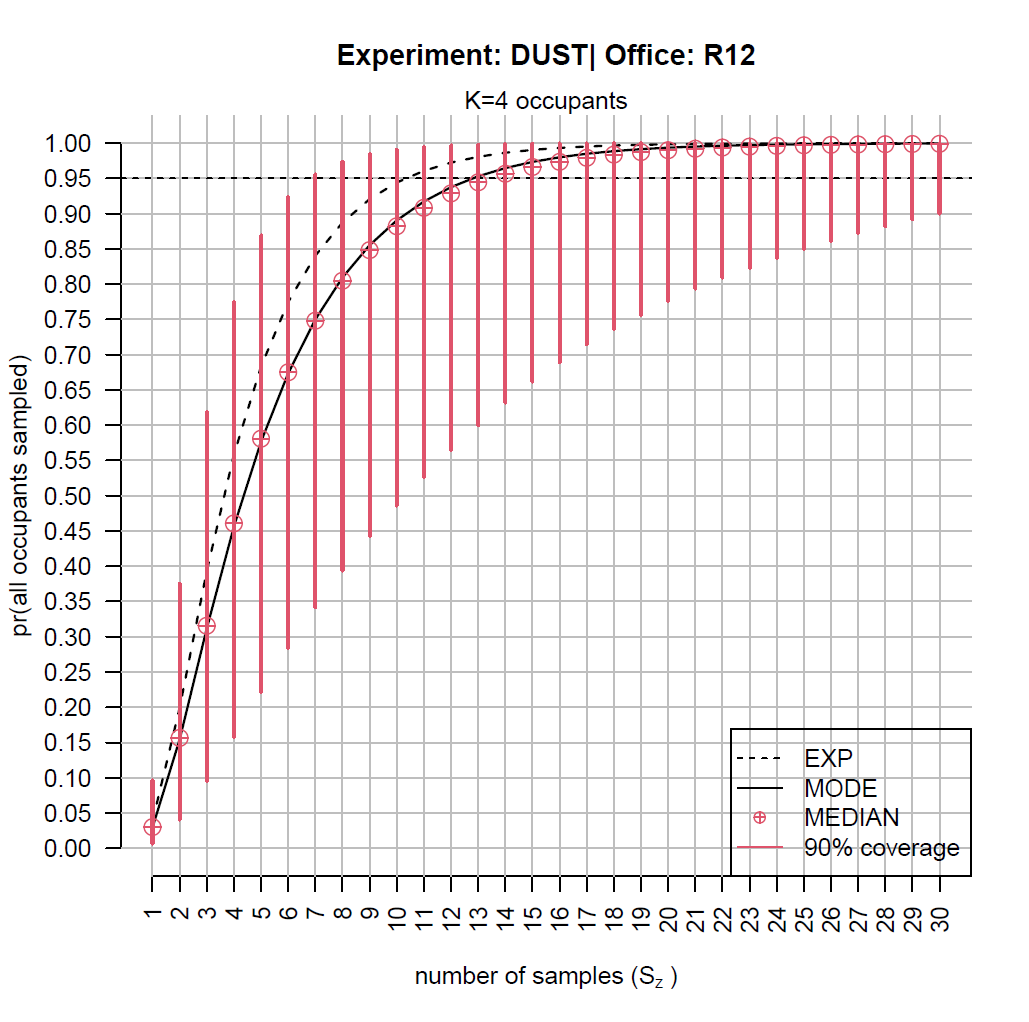


**Supplementary Fig. 11: Office R12, K=4: Plot of probability of detecting all k_z_ (**$\boldsymbol{\phi}_{\boldsymbol{z}}$**) at least once out of K occupants, in a set of samples, varying size (S_z_) using three different estimators.**


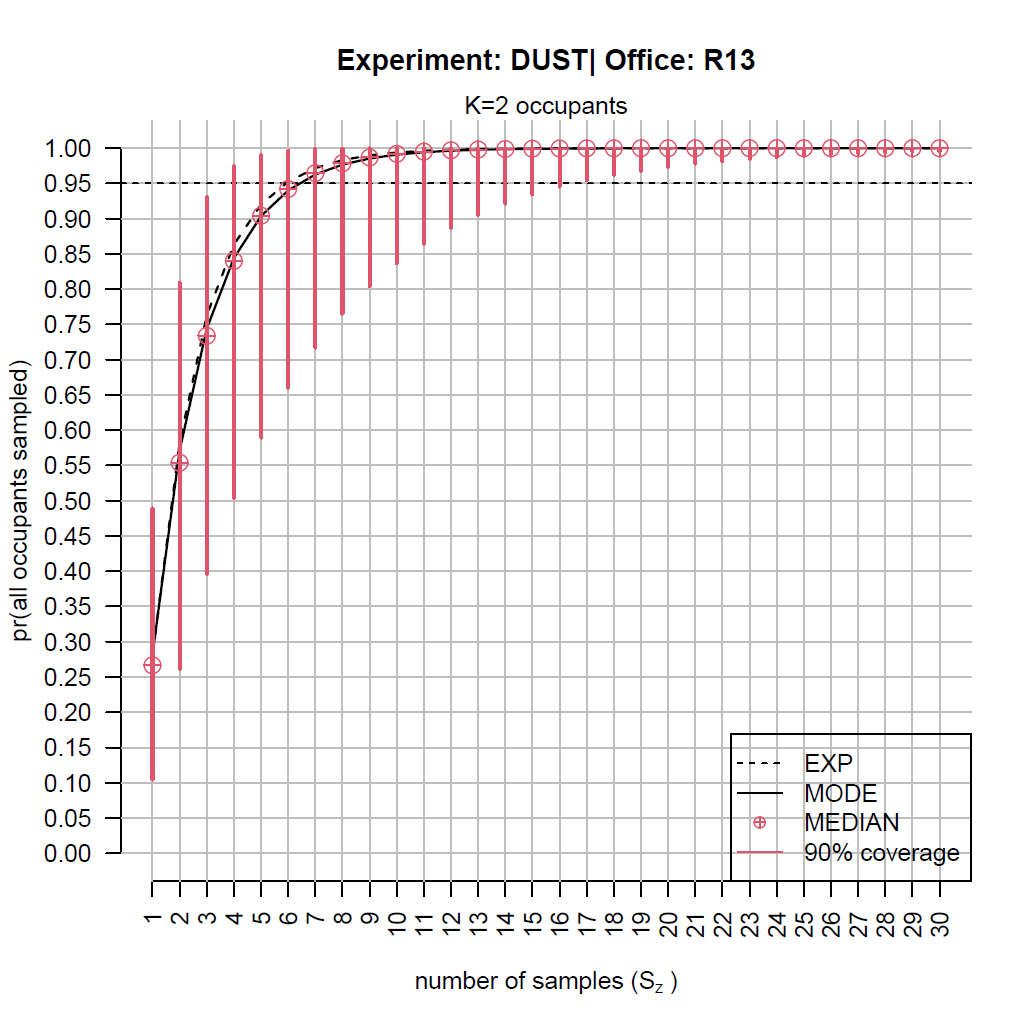


**Supplementary Fig. 12: Office R13, K=2: Plot of probability of detecting all k_z_ (**$\boldsymbol{\phi}_{\mathbf{z}}$**) at least once out of K occupants, in a set of samples, varying size (S_z_) using three different estimators.**


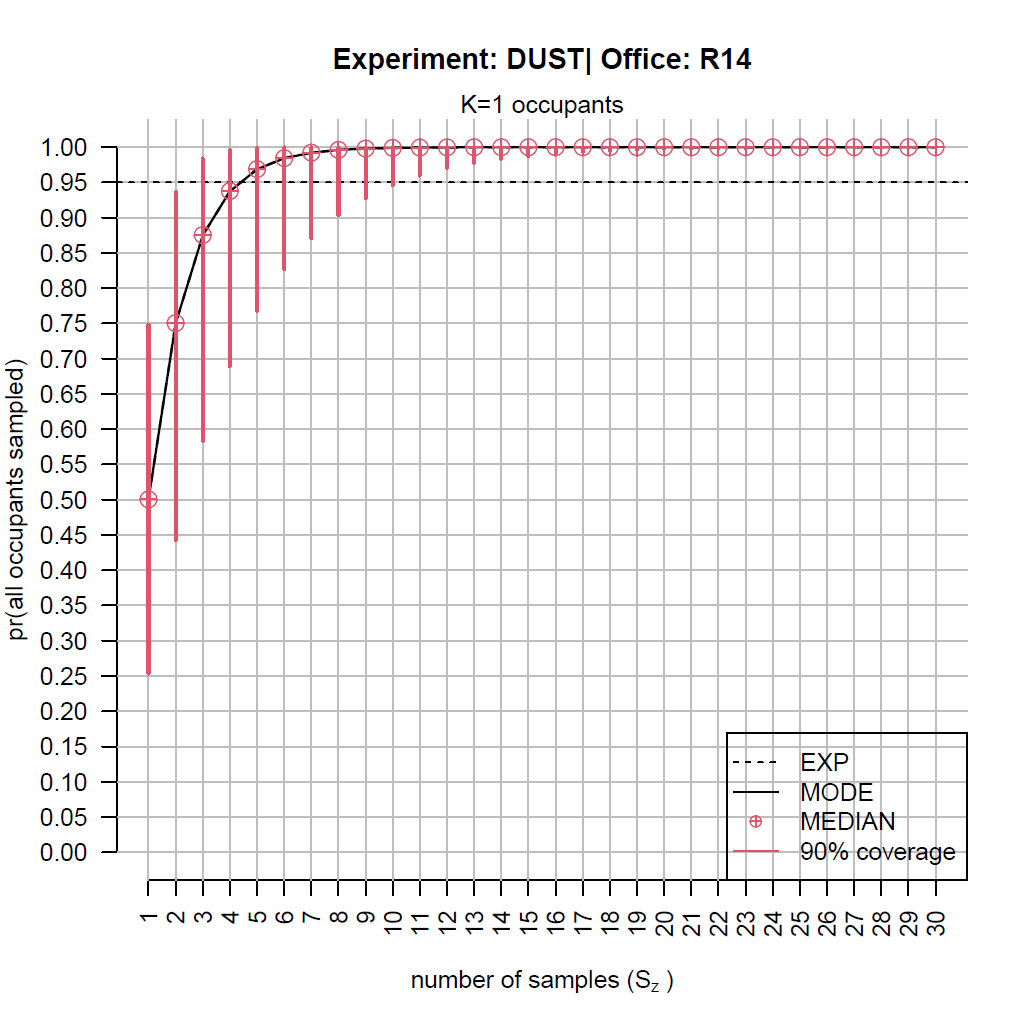


**Supplementary Fig. 13: Office R14, K=1: Plot of probability of detecting all k_z_ (**$\boldsymbol{\phi}_{\mathbf{z}}$**) at least once out of K occupants, in a set of samples, varying size (S_z_) using three different estimators.**
